# Reliability and validity of a novel tool to comprehensively assess food and beverage marketing in recreational sport settings

**DOI:** 10.1186/s12966-018-0667-3

**Published:** 2018-05-31

**Authors:** Rachel J. L. Prowse, Patti-Jean Naylor, Dana Lee Olstad, Valerie Carson, Louise C. Mâsse, Kate Storey, Sara F. L. Kirk, Kim D. Raine

**Affiliations:** 1 School of Public Health, University of Alberta, Edmonton, AB Canada; 2 School of Exercise Science, Physical and Health Education, University of Victoria, Victoria, BC Canada; 3 Department of Community Health Sciences, Cumming School of Medicine, University of Calgary, Calgary, AB Canada; 4 Faculty of Physical Education and Recreation, University of Alberta, Edmonton, AB Canada; 5 BC Children’s Hospital Research Institute, School of Population and Public Health, University of British Columbia, Vancouver, BC Canada; 6 Healthy Populations Institute, Dalhousie University, Halifax, NS Canada; 7 Centre for Health & Nutrition, University of Alberta, Edmonton, AB Canada

**Keywords:** Validity, Reliability, Food marketing, Food environment, Children and youth, Recreational sport settings

## Abstract

**Background:**

Current methods for evaluating food marketing to children often study a single marketing channel or approach. As the World Health Organization urges the removal of unhealthy food marketing in children’s settings, methods that comprehensively explore the exposure and power of food marketing within a setting from multiple marketing channels and approaches are needed. The purpose of this study was to test the inter-rater reliability and the validity of a novel settings-based food marketing audit tool.

**Methods:**

The Food and beverage Marketing Assessment Tool for Settings (FoodMATS) was developed and its psychometric properties evaluated in five public recreation and sport facilities (sites) and subsequently used in 51 sites across Canada for a cross-sectional analysis of food marketing. Raters recorded the count of food marketing occasions, presence of child-targeted and sports-related marketing techniques, and the physical size of marketing occasions. Marketing occasions were classified by healthfulness. Inter-rater reliability was tested using Cohen’s kappa (κ) and intra-class correlations (ICC). FoodMATS scores for each site were calculated using an algorithm that represented the theoretical impact of the marketing environment on food preferences, purchases, and consumption. Higher FoodMATS scores represented sites with higher exposure to, and more powerful (unhealthy, child-targeted, sports-related, large) food marketing. Validity of the scoring algorithm was tested through (1) Pearson’s correlations between FoodMATS scores and facility sponsorship dollars, and (2) sequential multiple regression for predicting “Least Healthy” food sales from FoodMATS scores.

**Results:**

Inter-rater reliability was very good to excellent (κ = 0.88–1.00, *p* < 0.001; ICC = 0.97, p < 0.001). There was a strong positive correlation between FoodMATS scores and food sponsorship dollars, after controlling for facility size (*r* = 0.86, *p* < 0.001). The FoodMATS score explained 14% of the variability in “Least Healthy” concession sales (*p* = 0.012) and 24% of the variability total concession and vending “Least Healthy” food sales (*p* = 0.003).

**Conclusions:**

FoodMATS has high inter-rater reliability and good validity. As the first validated tool to evaluate the exposure and power of food marketing in recreation facilities, the FoodMATS provides a novel means to comprehensively track changes in food marketing environments that can assist in developing and monitoring the impact of policies and interventions.

**Electronic supplementary material:**

The online version of this article (10.1186/s12966-018-0667-3) contains supplementary material, which is available to authorized users.

## Background

Scientific evidence indicates that unhealthy food marketing is a cause of childhood obesity [1]. A systematic review of over 100 studies found modest to strong causal evidence that unhealthy food promotion affected children’s food knowledge, preferences, purchases, consumption, and diet-related health [2]. Children around the world are exposed to food marketing that originates in and outside of their home country, thus protecting children from unhealthy food marketing is a local and international issue [3].

The World Health Organization (WHO) report of the Commission on Ending Childhood Obesity states that [4] “settings where children and adolescents gather (such as schools and sport facilities or events) …should be free of marketing of unhealthy foods and sugar-sweetened beverages” (p.18) as a means to reduce and prevent childhood obesity and promote optimal diets. One example of a place where children gather is recreation and sport facilities, which promote physical activity. Recreation and sport facilities represent ideal settings for population health interventions since thousands of children visit these sites to participate in physical activity [5]. Recreation and sport facilities are particularly crucial settings in which to measure food marketing because of the common food industry practice of emphasizing physical activity as a solution to obesity [6, 7]. Since many recreation and sport facilities are publicly funded, it is important that these settings offer and promote healthy food [8]. While modifications to the food environment in settings such as schools have received greater attention, there is increasing evidence that foods sold, marketed, and consumed by children in recreation and sport settings are not consistent with dietary guidelines [9–13].

The WHO called upon member states to measure the nature and extent of food and beverage marketing to children in their countries as a preliminary step to generating policy [3]. However, current methods of measuring food marketing to children do not capture all marketing channels through which children may be exposed to food marketing (i.e. television, internet, product packaging, placement) [14] and most were not designed to assess food marketing specifically in settings where children gather, such as recreation and sport facilities. In addition, no current methods collectively capture the impact of the four main marketing approaches: product, place, promotion and price (i.e. the 4Ps). The 4Ps represent marketing approaches that commercial and social marketers mix strategically to effectively persuade individuals to think or behave in a certain way [15]. Comprehensive measurement of the 4Ps across multiple marketing channels may reveal the intensity of food marketing that children may be exposed to in real life settings.

Without attention to the breadth and depth of potential food marketing channels and approaches in children’s settings, existing food environment assessment tools used in schools [16, 17], restaurants [18], and stores [19–21] fall short of capturing a full picture of the food marketing environment. First, some tools only measure a single marketing approach: Velazquez et al. [16] measured only promotions (one of the 4Ps) in schools; Hosler et al. [20] only measured one marketing channel (exterior window advertisements) in stores. On the other hand, other tools collect minimal details on multiple marketing approaches perhaps because measuring food marketing was not the primary purpose of these tools. For example, in restaurants, Saelens et al. [18] used only a few questions to measure place, price, and promotion. In stores, Laska et al. [21] measured place and promotion only. Furthermore, some tools dichotomize the presence or absence of food marketing without considering the intensity of marketing in an area [17, 19, 21]. Finally, only a few tools have been tested for reliability [16, 18–20] and none have been tested for validity.

To better understand the nature and extent of food marketing within settings where children gather, we developed a theoretically grounded, evidence-informed observational Food and beverage Marketing Assessment Tool for Settings (FoodMATS). The FoodMATS provides a novel method to measure food marketing by gathering and scoring detailed information on numerous food marketing approaches and channels children may be exposed to. This study tested the inter-rater reliability of the FoodMATS indicators and evaluated validity of its scoring algorithm by evaluating convergence between FoodMATS scores and facility sponsorship dollars, and between FoodMATS scores and unhealthy food sales.

## Methods

### Setting, participants & measures

Data were collected as part of the Eat Play Live (EPL) study investigating food environments in public recreation facilities in four provinces in Canada. To be eligible to participate facilities must: (1) have provided food services through vending or concession (such as a canteen, snack bar, café, or restaurant), (2) had not made major changes to their food environment since 2010, (3) be able to make changes to their food environment (as the facility may be randomly assigned to a capacity-building intervention to improve food environments, not discussed here), and (4) had year-round sport programming. Recreation facilities were recruited between August 2015 and May 2016 by provincial partners through newsletters, email, and conference sessions; the EPL research team followed up with managers of facilities within proximity of universities only due to logistical constraints of the above mentioned planned intervention (not discussed here) by telephone and/or personal emails.

Forty-nine of the 286 facilities contacted by the EPL team agreed to participate. Of the remaining, 141 did not respond to the invitation; 70 were not eligible; 11 refused without reason; 15 refused with reason [insufficient staff capacity (*n* = 11), uninterested in research (*n* = 2), risk of being a control site (*n* = 1), worried about competition (n = 1)]. Food and beverage marketing was measured in 51 sites (two facilities operated two buildings each that were geographically separated; we treated each building as an individual site rather than combining the sites since a patron would usually only visit one site at a time, resulting in 51 sites from 49 facilities). The sample size was determined by a priori power calculations with G*Power (v3.1), which determined that at least 43 sites were required to detect a medium-large effect in the availability of healthy and unhealthy foods and beverages in vending machines with 80% power.

### FoodMATS development of tool and scoring algorithm

The FoodMATS was developed to capture overall exposure to food marketing in recreation facilities, what products (tangible food or beverage item), brands (name or symbol that represents the maker of a product), and retailers (place where food can be purchased, such as a store or restaurant) were marketed, where food marketing was placed, and whether persuasive marketing techniques were used. The scope and content of the FoodMATS was informed by previous research measuring: (a) food marketing by marketing channel [14]; (b) food marketing within schools [16, 17], restaurants [18], and stores [19–21]; (c) food marketing targeted to children [2, 22] and (d) sports-related food environments and marketing [23, 24].

Two conceptual models from business [25] and population health [26] informed the content and scoring of the FoodMATS. First, the 4Ps marketing mix [25], was used to identify the breadth of marketing approaches to be assessed by the FoodMATS that may be present in a recreation facility (Table 1). Secondly, the WHO’s *Exposure and Power of Marketing Messages* model [26] informed the depth of information collected by the FoodMATS (Table 2). This model explains how the impact of food and beverage marketing to children on food preferences, purchases, and consumption depends on the exposure and power of marketing messages, where exposure is “the reach and frequency of the marketing message”, and power is “the creative content, design and execution of the marketing message” (p.11). As the WHO model provides only broad definitions of exposure and power, we developed our own evidenced-based operational definitions for the FoodMATS.

**Table 1:** FoodMATS Operational Definitions of 4Ps Marketing Mix

Product	• Food or beverages available for purchase in concessions or vending machines or the food or beverage product, brand, or retailer marketed in the recreation facility (whether or not it was available within the recreation facility). • Classified as “Most Healthy”, “Less Healthy”, or “Least Healthy” foods or beverages (Table 2).
Price	• Monetary cost of food and beverages available in vending machines and concessions located within the recreation facility. • The FoodMATS includes 11 pricing indicators: four were related to overeating or rewards for repeat visits; seven compared prices of healthy and unhealthy food and beverage options. • Pricing indicators were classified as “Least Healthy” if pricing encouraged overeating, repeat visits, or unhealthy options (e.g. sugar sweetened drinks) were cheaper than healthy options (e.g. water).
Place	• Physical location of where food and beverages are placed or marketed. • The FoodMATS includes 26 locations (e.g. windows, scoreboards, checkouts) where food marketing may be found in the recreation facility. • All locations were grouped into three facility areas: food (concession), sports, and other (entrance, hallways, outside).
Promotion	• Advertising, messaging, or communication to persuade recreation facility users to purchase, use, or consume any food or beverage or to increase brand awareness. • Used by raters to identifying the presence and count of food marketing occasions.

**Table 2:** FoodMATS Operational Definitions of Exposure and Power of Marketing

Exposure	Frequency	Number of food or beverage marketing occasions. A marketing occasion was defined as any commercial advertising, promotion, or messaging of food or beverage products, brands, retailers (i.e. restaurant) that is intended to increase the “recognition, appeal and/or consumption” of such products/ brands [26] (p.9). Excludes product packaging.	
	Repetition	Number of products, brands, retailers that are recorded three or more times per facility during the observational audit.	
Power	Content	Healthfulness of product, brand, or retailer that is promoted. Classified by ordered categories for products/brands, and retailers.	
		Product/Brands: “Most Healthy” = unprocessed food/beverages with no added fat, sugar or salt; “Less Healthy” = some added fat, sugar, or salt; “Least Healthy” = processed energy-dense, nutrient-poor items with high levels of fat, sugar, or salt.	Retailers: “Most Healthy” = grocery stores, farmers’ markets, salad bars, sandwich outlets, smoothie outlets; “Less Healthy” = sit-down restaurants, cafeterias, coffee outlets, prepared grocery stores, supplement stores; “Least Healthy” = pizza, burger, taco, fried chicken, Asian, and ice cream outlets, pubs/lounges/alcohol stores.
	Design	Use of child-targeted techniques and/or inclusion of sports-related theme in promotion.^a^ Recorded as present or absent. Child-targeting techniques were those that had evidence of animated or fictional characters, taste appeals, humour, action-adventure, fantasy, fun (shapes, colours), competitions, give-aways, [2] cartoonish font [22], or that used a child actor^b^ to advertise a food or beverage product/brand that would appeal to children. Sports-related techniques were those that had any reference to physical activity, exercise, sport, game, recreation, performance or competition^c^.	
	Execution	Physical size of the promotion.^a^ Recorded as ordered categories, using different size requirements for outdoor and indoor marketing occasions.	
		Outdoor [19]: Small < 1 letter sheet piece of paper (8.5 X 11 in); Medium 1–10 letter size sheets of paper together; Large > 10 pieces of letter size sheets of paper together	Indoor [16]: Small < 1 letter sheet piece of paper (8.5 X 11″); Medium 1–3 letter size sheets of paper together; Large > 3 letter size sheets of paper together

^a^ Excluded for some pricing and place marketing occasions

^b^ added post pilot after this technique was identified

^c^a design feature relevant to sport settings

We used the count and repetition of food marketing to represent ‘exposure’ of food marketing (Table 1). Based on previous research suggesting that certain marketing techniques have unique or strong impacts on food choice (described below), we used the healthfulness of the product, brand, retailer marketed, use of child-targeted and sports-related marketing techniques, and size of each marketing occasion as FoodMATS indicators to represent the ‘power’ of food marketing (Table 1).

Unhealthy food marketing is considered ‘powerful’ since children have an innate desire for nutrient poor foods and immediate gratification and are thus less able to resist unhealthy food marketing [27]. Experimental research found that children preferred less healthy foods and beverages over more healthy options even when the more healthy option was marketed to them with licensed characters [28].

Targeting of children in food marketing through characters, appeals of taste, humour, action-adventure, fantasy and fun, and incentives (giveaways) are common practices worldwide [2] and should be monitored [14]. We considered marketing techniques that target children ‘powerful’ since children’s cognitive immaturity makes them vulnerable to the effects of marketing [29]. Child-targeted marketing techniques, such as fun product packaging [22], toy premiums [30], and games [31] have been shown to impact children’s and parents’ desire to consume and purchase advertised foods.

Sports-related food marketing techniques, such as using themes of physical activity or exercise, are also considered ‘powerful’ because they have shown to impact product perceptions in adults and children [32–34]. For example, children who reviewed a commercial for sugary cereal that contained some aspect of physical activity had more positive reactions to the cereal and believed the cereal to be healthier than the children who viewed the cereal commercial with no reference to physical activity [32].

Finally, size is considered a ‘powerful’ feature since marketing eye tracking research found that the larger the advertisement, the more attention the viewer paid to it [35].

The final FoodMATS included 37 marketing items, including 26 locations and 11 pricing indicators (Table 1). A copy of the FoodMATS tool can be found in Additional file 1. For each marketing item, the rater recorded:

- the presence of food or beverage marketing occasions by item;
- the count of food and beverage marketing occasions by area;
- the product, brand, retailer identified in the marketing occasion;
- whether the marketing occasion was child-targeted;
- whether the marketing occasion was sports-related; and
- the size of the marketing occasion.

During data collection, raters used a priori definitions to classify each marketing occasion as child-targeted, and/or sports-related, as well as its physical size (Table 2). After data collection, we classified marketing occasions by healthfulness (described in Data Collection).

The exposure and power of food marketing recorded were used to derive a FoodMATS score for each site. Points were assigned for the frequency of observed food marketing occasions and the proportion of marketing occasions with ‘powerful’ characteristics in each area (food, sport, other). Site scores were generated by summing area scores and adding a repetition factor for the number of products, brands, or retailers marketed repeatedly in the entire site (see Additional file 2 for more information on scoring). Higher scores represent settings with greater exposure and more powerful food marketing which, according to the WHO’s *Exposure and Power of Marketing Messages* theory [26], may identify environments that may be more harmful on children’s food preferences, purchases, and consumption.

### Data collection

#### Inter-rater reliability testing

Inter-rater reliability of the FoodMATS was tested by five raters. Five urban public recreation facilities that offered food through vending machines and/or concessions were selected for testing in October–November 2015. Facilities of different sizes and sport offerings were selected to investigate the use of the FoodMATS in different types of recreation and sport settings. Two independent trained raters completed the FoodMATS at the same facility on the same day and photographed each food marketing occasion.

#### EPL baseline – validity testing

Following inter-rater reliability testing, the FoodMATS was completed in 51 EPL sites between December 2015 and April 2016 by a trained rater. Food and beverage marketing was photographed and recorded in food (concession) areas, sports areas, and other general areas (entrance, hallways, bathrooms, parking lot) of the site. Specialty areas (i.e. theatres, day cares, meeting rooms, etc.) were not audited. All marketing occasions recorded were checked by the first author (RJLP) against photos taken to confirm marketing frequency, the product, brand, retailer marketed, use of child-targeting and sports-related marketing techniques, and size. Inconsistencies were resolved via a consensus process with the rater and the first author, including another investigator (KDR) if necessary.

One registered dietitian (RJLP) independently classified the healthfulness of every food and beverage product, brand, and retailer recorded in the FoodMATS for all 51 sites, which was checked by a second registered dietitian (KDR). We used ordered classes to rank food and beverage products, brands, and retailers (“Most Healthy”, “Less Healthy”, or “Least Healthy”) (Table 2) which paralleled provincial nutrition guideline categories [36–39] which assess food and beverage healthfulness according to nutrient and ingredient content per reference size (see Additional file 3); we could not use exact provincial guideline categories due to lack of detailed nutrient information for many products marketed in recreation facilities. Given that it was not feasible to collect and analyze nutrient content of all products marketed, several simplifying assumptions were made for the purposes of classifying products as more or less healthy (see Additional file 4). If needed, the Canadian Nutrient File (https://food-nutrition.canada.ca/cnf-fce/index-eng.jsp) or product company websites were used to obtain more information about foods and beverages. Brands were ranked as per the product rankings described above for the product the brand most closely represented (e.g. Coca-Cola is known for sweetened soft drinks; Aquafina is known for water). The healthfulness of food retailers was assessed according to rankings of healthfulness of food retailers by Minaker et al. [40] which were ranked based on their relative availability of healthy food and preparation methods. When retailers not evaluated by Minaker et al. [40] were recorded on the FoodMATS, we placed retailers into the three categories as per their most prominent product sold based on the retailers’ name and menu (e.g. fried chicken for a fast food retailer called Mary Brown’s Chicken & Taters, ice cream for a fast food retailer called Dairy Queen). Each site was assigned a FoodMATS score based on the exposure and power of food marketing recorded.

Two weeks of food and beverage sales data that did not include an unusual day (e.g. tournament or site closure) were requested from all vending and concession operators from all 51 sites. Foods and beverages recorded on concession sales data were classified with the same ordinal classification scheme described for the FoodMATS products by two registered dietitians (RJLP, AH); any disagreements were resolved by a third dietitian (KDR). Since detailed product nutrient information was available for items in vending machines from a public database, Brand Name Food List (https://bnfl.healthlinkbc.ca/), provincial nutrition guidelines from each site’s respective province was used to classify products (except for vending machines in the non-guideline province). Products in vending machines from the non-guideline province were classified according to British Columbia’s provincial nutrition guidelines. Specifically, foods and beverages classified as “Do Not Sell” in “British Columbia [38] and Ontario,” “Choose Least Often” in Alberta [36], and “Minimum” in Nova Scotia [39] represented “Least Healthy” vending sales. Total “Least Healthy” sales equaled the sum of “Least Healthy” sales from concession and vending for sites that had data for both available (when applicable). We adjusted all concession, vending, and total sales to represent 1week of sales per site. Based on the *Exposure and Power of Marketing Messages* model, marketing is expected to impact food preferences, purchases, and consumption [26], therefore we hypothesized that FoodMATS scores should explain some variability in unhealthy food sales.

Total and food-related sponsorship dollars facilities that were received by facilities during the 2015/2016 fiscal year were requested from a subset of 27 volunteer sites in two provinces (BC, AB). We defined sponsorship dollars as dollars paid by outside companies to support facility operations and/or to advertise in and around a facility. Food-related sponsorship dollars were dollars provided by food retailers. Since sport sponsorship is usually combined with on site ads, signs, and displays [41], we hypothesized that higher FoodMATS scores would be correlated with higher food-related sponsorship dollars.

### Data analysis

#### Inter-rater reliability

Data were entered and cleaned in Microsoft Excel 2013. Statistical Package for the Social Sciences Version 23 (SPSS Inc., Chicago, IL, USA) was used for all statistical analyses with *p <* 0.05 indicating statistical significance. Agreement between the two raters for each site was assessed based on whether raters agreed food marketing was present or absent per item and the count of marketing occasions per area (food, sport, other). For marketing occasions that were identified by both raters, we tested whether raters agreed on what product, brand, or retailer was marketed, and whether the marketing occasion was children-targeted and/or sports-related, and its physical size.

Percent agreement [42] for these items was calculated by determining the proportion of occasions of perfect agreement out of all possible occasions. Cohen’s kappa (κ) was used to determine agreement between raters on categorical data (unweighted κ for nominal data; weighted κ_w_ for ordinal data). The interpretation of Cohen’s kappa was as follows: 0.0–0.2 fair, 0.21–0.40 poor; 0.41–0.60 moderate; 0.61–0.80 good; 0.81–1.00 very good agreement [43]. Intra class correlations (ICC) were used to determine consistency between raters for continuous data [44, 45]. Continuous data were square root transformed to improve normality. Two-way random ICC [45] were completed on the transformed data. The ICC interpreted as follows: < 0.40 poor; 0.40–0.59 fair; 0.60–0.74 good; 0.75–1.00 excellent [46]. The ICC for using the measure with 1 rater are reported.

#### Validity

FoodMATS scores, total and food-related sponsorship dollars, and weekly sales of “Least Healthy” foods and beverages for concessions, vending, and in total were entered into Statistical Package for the Social Sciences Version 24 (SPSS Inc., Chicago, IL, USA) for analysis with *p* < 0.05 indicating statistical significance.

First, validity of FoodMATS scores was tested by using Pearson’s Product Moment correlations, which correlated the FoodMATS scores with total and food sponsorship dollars. To improve normality, FoodMATS scores and food sponsorship dollars were transformed by taking the square root of the data. One outlier was truncated for FoodMATS score and food sponsorship dollars to one point above the next closest value below 3 standard deviations in the data set to reduce its effect [47, 48]. We ran partial Pearson’s Product Moment correlations controlling for site size, defined as the number of concessions and number of sports areas per site.

Next, validity of FoodMATS scores was tested using by sequential multiple linear regression to examine associations between the dependent variables (concession, vending, and total “Least Healthy” sales) and the explanatory variable (FoodMATS). Site size (as defined above) was entered as Model 1 as a controlling variable, then FoodMATS scores were added to site size for Model 2. We square root transformed the “Least Healthy” sales which resulted in normal distributions of the residuals.

We used “Least Healthy” sales because the majority of marketing occasions were “Least Healthy” and FoodMATS scores increased with greater proportions of “Least Healthy” marketing occasions. Additionally, the availability of “Most Healthy” products for sale was very low relative to “Least Healthy” products in most sites, possibly obscuring relationships between marketing and sales of “Least Healthy” products due to limited availability.

To assess the impact of missing data, independent t-tests were used to assess if there were differences in the mean FoodMATS scores between sites that provided sponsorship and sales data and those that did not.

## Results

### Reliability

Inter-rater reliability results can be found in Table 3. Percent agreements were high for all components evaluated except for identifying the same count of marketing occasions by area (61%). However, the ICC for identifying the same count of marketing occasions per area was excellent. Raters also had very good agreement on identifying the presence of marketing. For marketing occasions identified by both raters, there was very good agreement for identifying the product, brand, retailer marketed, the presence of child-targeted and sports-related features, and its size.

**Table 3:** Inter-rater reliability statistics from pilot testing FoodMATS

Reliability Component	n	Percent Agreement	Inter-rater reliability coefficients
(a) Presence of food marketing by item	464	92.2%	κ = 0.875 (95% CI 0.847, 0.903)***
(b) Count of food marketing occasions by area	28	61.1%	ICC (2, 2) = 0.934 (95% CI (0.808, 0.978)***
(c) Product marketed^a^	218	100.0%	κ = 1.00 (95% CI 1.000,1.000)***
(d) Child-targeted marketing^a^	184	100.0%	κ = 1.00 (95% CI 1.000,1.000)***
(e) Sports-related marketing^a^	184	98.9%	κ = 0.941 (95% CI 0.883, 0.999)***
(f) Physical Size^a^	180	92.2%	κ _w_ = 0.911 (95% CI 0.846, 0.976)***

*** *p* < 0.001

^a^ when both raters identified that food marketing was present

### FoodMATS score validity

Median and interquartile ranges of sponsorship, sales, facility size, and FoodMATS scores can be found in Table 4.

**Table 4:** Descriptive statistics of “Least Healthy” food and beverage sales and FoodMATS scores

Variable	N	Median	Interquartile Range^a^
Facility Sponsorship Dollars			
Total Sponsorship ($)	16	15,452.50	7630.50, 32,825.00
Food Sponsorship ($)	18	1350.00	0.00, 4120.50
“Least Healthy” Food and Beverages Sales			
Total Sales ($)	21	1100.35	290.32, 2521.94
Concession Sales ($)	30	1515.94	466.82, 2354.15
Vending Sales ($)	23	280.53	121.00, 567.58
Facility Size			
Concessions (n)	51	1	1, 1
Sports Areas (n)	51	3	2, 5
Marketing Scores			
FoodMATS (points)	51	43.3	18.6, 71.0

^a^ 25th percentile, 75th percentile

#### Association with sponsorship dollars

Sixteen facilities (64.0%) provided the total sponsorship dollars received annually. Eighteen facilities (72.0%) provided food sponsorship dollars received annually. FoodMATS scores were linearly correlated with food sponsorship dollars (*r* = 0.900, *p* < 0.001) but not with total sponsorship dollars (*r* = 0.390, *p* = 0.136), thus no further analysis with total sponsorship was completed. There was a strong positive correlation between FoodMATS and food sponsorship dollars (*r* = 0.815, *p* < 0.001; rho = 0.842, *p <* 0.001). After controlling for facility size, the correlation between FoodMATS and food sponsorship dollars remained strong (*r* = 0.863, *p* < 0.001). There were no differences in mean FoodMATS scores between sites that provided food sponsorship dollars and sites that did not (*p* = 0.895).

#### Predicting sales of less “healthful” food and beverage items

Thirty-four concessions (70.8%) provided concession sales for 2 weeks. Four concessions were excluded due to poorly itemized sales data which inhibited classification of products sold by healthfulness, resulting in 30 sites for the final sample size for concession sales. Thirty-seven sites (75.5%) provided vending sales data. Data from 14 sites were excluded (seven had poorly itemized sales data which inhibited classification of products sold by health; seven provided incomplete sales data), resulting in a final sample size for analysis of vending sales from 23 sites. Twenty-one sites (41.2%) had complete sales data for vending and concessions. There were no differences in mean FoodMATS scores between sites that did and did not provide concession (*p =* 0.881), vending (*p =* 0.563), and total sales (*p =* 0.726).

In the initial regression analysis, FoodMATS scores and number of concessions were highly correlated (*r* > 0.7) and the number of concessions was not predictive of “Least Healthy” sales in the concession (*r* = 0.224, *p* = 0.097) so we excluded number of concessions as a predictor of FoodMATS scores [49] and re-ran the regression models. Regression results can be found in Table 5. Model 1 (facility size defined as the number of sports areas) significantly predicted “Least Healthy” sales in concessions, vending, and in total. Model 2 (FoodMATS scores and facility size) did not significantly predict “Least Healthy” sales in vending, but significantly predicted “Least Healthy” sales in concessions and in total; explaining 45.1% and 42.8% of the variance of “Least Healthy” sales in concessions and in total, respectively. The FoodMATS score significantly explained an additional 13.8% of the variability in sales of “Least Healthy” items in concessions (*F* change (1, 27) = 7.300, *p =* 0.012) and 23.5% of the variability in total sales (*F* change (1, 18) =8.485, *p =* 0.003).

**Table 5:** Sequential multiple regression analyses predicting square root transformed weekly sales of “Least Healthy” ^a^ foods and beverages from FoodMATS scores and facility size

Predictor		Beta^a^ (95% confidence interval)	Beta^b^ (95% confidence interval)	*R*^2^ (adjusted)	*R*^2^ change (adjusted)	F
Concession sales (*n* = 30)						
Model 1:	*Facility Size*			0.328**	0.351**	15.149**
	Number of Sports Areas	0.593** (2.42–7.79)	0.517** (1.97–6.94)			
Model 2:	*Marketing Scores*			0.451***	0.138*	12.929***
	FoodMATS Score		0.379** (0.03–0.24)			
Vending sales (*n* = 23)						
Model 1:	*Facility Size*			0.184*	0.221*	5.960*
	Number of Sports Areas	0.470* (0.37–4.66)	0.448* (0.17–4.63)			
Model 2:	*Marketing Scores*			0.156	0.012	3.038
	FoodMATS Score		0.111 (−0.07–0.12)			
Total (concession and vending sales) (*n* = 21)						
Model 1:	*Facility Size*			0.210*	0.250*	6.329*
	Number of Sports Areas	0.500* (1.12–12.16)	0.505** (1.98–11.42)			
Model 2:	*Marketing Scores*			0.428**	0.235*	8.485**
	FoodMATS Score		0.485* (0.04–0.29)			

^a^ Standardized regression coefficients without marketing scores entered into the regression

^b^ Standardized regression coefficients with marketing scores entered into the regression

*p < 0.05. **p < 0.01. ***p < 0.001

We tested the robustness of the regression results by evaluating whether food marketing outside of the concession, FoodMATS scores from non-food areas (Sports, and Other), predict “Least Healthy” sales in the concession. Sport area FoodMATS scores significantly predicted “Least Healthy” sales in concessions (β = 0.285, 95% CI 0.085–0.485, *p* = 0.007). Other area FoodMATS scores also significantly predicted “Least Heatlhy” sales in concessions (β = 0.643, 95% CI 0.111–1.175, *p* = 0.020). On the other hand, Food area FoodMATS scores on their own were not associated with (*r* = 0.294, *p* = 0.057), suggesting that evaluating food marketing in the whole setting, not just food areas, is critical to understand how food marketing in recreation and sport facilities may impact food and beverage sales.

## Discussion

The FoodMATS tool performed well in both reliability and validity analyses. These findings suggest that individual raters collected very similar data when completing the FoodMATS and the scores assigned to each site represent constructs of the food marketing environment related to exposure, power, and impact.

### Reliability

Measures of inter-rater reliability were very good to excellent. The measures of reliability were chosen to reflect how the information would be interpreted for scoring which means that the consistency between raters identifying marketing frequency and characteristics should translate to consistency in FoodMATS scores. Providing specific operational definitions of marketing components and adequate training may have contributed to these positive results.

The percent perfect agreement may have been lower for the count of marketing occasions per area than for other reliability measures as raters may have different interpretations of what one occasion meant. For example, one rater may interpret three of the same beverage logos on a vending machine as three marketing occasions, whereas the other rater may record that as one. Despite the low percent perfect agreement, the ICC for the count of marketing occasions per area was excellent suggesting that even though raters did not always count the exact same number of marketing occasions their counts were close. For example, rater 1 may have counted 17 marketing occasions in one area and rater 2 counted 18.

The inter-rater reliability of the FoodMATS is comparable to other settings-based food environment tools, including the Nutrition Environment Measures Survey in Restaurants (NEMS-R) [18], and in grab-and-go establishments (NEMS-GG) [50]. In a study documenting food and beverage promotions in schools, Velazquez et al. [16] had almost perfect inter-rater reliability for most items, similar to this study. The FoodMATS tool had better inter-rater reliability than a tool measuring number of healthy and less healthy outdoor store promotions (κ = 0.37–65) and presence of healthy and less healthy advertisements or products at store checkouts (ICC = 0.466–0.697) [19]. The FoodMATS may have performed superiorly because each marketing feature was assessed individually, whereas Ghirardelli et al. [19] combined multiple constructs (frequency, size, and healthfulness) into one item when documenting marketing and assessing inter-rater reliability.

### FoodMATS scores validity

#### Association with sponsorship dollars

Total sponsorship and FoodMATS scores were not linearly related as there were some sites that received high amounts of sponsorship funding from third parties that had low FoodMATS scores (lower exposure to food marketing and/or less powerful marketing), and other sites had low amounts of sponsorship funding with high FoodMATS scores (greater exposure to food marketing and/or more powerful marketing). On the other hand, the sponsorship dollars that facilities received from food-related companies were significantly correlated with FoodMATS scores. The lack of correlation between total sponsorship and FoodMATS scores and strong correlation between food sponsorship and FoodMATS scores may indicate that the FoodMATS scores truly represent food marketing in the facility, and not marketing in general.

As the FoodMATS and its scoring algorithm is a novel tool to measure and classify food and beverage marketing in settings, there is little research to compare the results to. However, previous related research may help to explain results. In Australia, only 17% of all sports club sponsors were food and beverage companies [24], which may explain why there was no linear relationship between FoodMATS scores and total sponsorship. In our study, food sponsorship dollars contributed a median of 12.0% (IQR: 3.9, 25.6%) of total sponsorship dollars for the 11 sites that provided both food and total sponsorship dollars (data not shown).

The high correlation between FoodMATS and sponsorship dollars may be surprising because the FoodMATS collects several marketing items that may or may not be part of a sponsorship agreement. However, Kelly et al. [24] found food and beverage sponsors of sports clubs in Australia engaged in numerous marketing activities besides direct funding to show their support of the club or sport, including uniform branding, being official club sponsors, naming in newsletters, signage, offering sponsor’s product, and providing rewards.

#### Predicting sales of “less healthy” food and beverage items

FoodMATS scores significantly explained almost half of “Least Healthy” food and beverage sales in concessions and in total. The large effect size of the FoodMATS score on total “Least Healthy” sales suggests that greater exposure and/or powerful food marketing in recreation facilities contributes to higher “Least Healthy” sales, in line with the theoretical underpinning of the scoring algorithm. The lack of prediction of vending sales by FoodMATS may be related to a small sample size or low sales since vending sales only contributed an average of less than one-third of total facility sales when both concession and vending are present (data not shown).

The prediction of “Least Healthy” food sales in concessions from FoodMATS is interesting, especially from marketing outside of concessions in sport and other areas, because it may represent that more food marketing throughout a facility results in more traffic to the concessions to purchase food. A recent meta-analysis found that there is an immediate modest impact of unhealthy food marketing on children’s food intake [51]. In the context of this setting, that may mean that children who see food marketing in a recreation facility may eat more and some of the food consumed may be purchased on site. Furthermore, findings from a systematic review suggest that food marketing impacts food purchases at the brand and category level [2]; thus, it is possible that unhealthy food marketing in recreation facilities could impact food sales in general regardless of whether the exact product or brand marketed was available for purchase on site. These findings provide support for settings-based measurement to fully understand the extent of children’s food marketing environments.

Previous research has shown healthy food availability to be associated with greater purchases of the same [23, 52]. Future research should investigate the interrelationship between food availability, marketing, and sales to best provide recommendations on how to generate a health promoting food environment within recreation facilities while maintaining profitability.

### Limitations

The FoodMATS may not capture certain types of non-permanent food marketing, such as team sponsorship, giveaways, or fundraising; nor does it capture marketing on product packaging. As well, the analysis of the FoodMATS data did not include content analysis of the promotions, thus providing limited information on types of marketing techniques used. We excluded the placement height of marketing as an indicator child-targeted marketing since vending machine, concessions, or other areas may only have a certain amount of space causing them to place items or promotions at child height without intentionally marketing to them. This exclusion may underestimate child-targeted marketing.

Due to limited product nutrient information, we were unable to assess nutrient content of products by the WHO Regional Office for Europe Nutrient Profiling Model which was developed to inform marketing to children restrictions. This profiling model should be considered in future use of the FoodMATS when product nutrient information can be collected. Nevertheless, the provincial guidelines used in this study to assess healthfulness were highly relevant to our local context.

It is possible that 2 weeks of food and beverage sales data may not represent facilities’ overall sales which could have impacted the regression results. Although a relative outcome variable, such as the ratio of “Most Healthy” to “Least Healthy” sales, rather than the absolute variable we used may be more fitting to test the validity of the FoodMATS score, we were unable to use the former because there was little variability in the proportion of sales that were “Least Healthy”. Also, we were only able to evaluate the relationship between the food marketing environment and sales at the site level, which cannot be interpreted at the individual level. The results presented here should be interpreted with some caution, since the sites selected for EPL are not necessarily representative of all recreation and sport facilities in Canada and the small sample sizes limit the power of analyses.

### Strengths

No other research tool measures marketing as comprehensively as the FoodMATS or include a scoring algorithm that quantifies the potential negative impact of a food marketing environment. Diverging from the previous self-report survey methods used to measure food and beverage marketing in recreation and sport settings [53], the FoodMATS collects detailed data grounded in theory relevant to public health and business practitioners. Investigating the relationship between food marketing environments and sales to validate the FoodMATS scores in our unique study presents a new avenue to advance researchers’ abilities to study the impact of food marketing environments on diet. To understand broader, whole setting-based influences on dietary habits future research could assess relationships between FoodMATS scores and healthy food sales, changes in FoodMATS and sales, individual level purchases, and purchases by different demographic groups.

Although the FoodMATS was designed for sport settings, it could be adapted for other settings, such as schools, enabling comparison across settings where children gather. The tool has only been used with trained research staff, but it is possible that it could be used with trained community members to conduct self-assessments. Additional supports may be necessary to enable community use such as an online system where marketing can be entered and automatically scored. Most importantly, the FoodMATS tool can be used to inform and evaluate regulatory interventions aimed at reducing children’s exposure to powerful unhealthy food marketing.

## Conclusions & Implications

In 2010, the World Health Assembly (WHA) endorsed the WHO set of recommendations on the marketing of foods and non-alcoholic beverages to children but was met with insubstantial follow through by member states [54]. Last year, the WHA recommitted to action by supporting the report of the Commission on Ending Childhood Obesity which includes implementing the WHO’s food marketing recommendations [55]. Understanding the landscape of food marketing to children within settings where children spend time is important in order to generate effective policy interventions that will reduce children’s exposure to marketing and the power of that marketing [26]. The FoodMATS is a novel audit tool that can be reliably used to analyze food marketing in children’s recreation and sport settings. Its scoring algorithm has good validity and can therefore be used to explore the unhealthfulness of food marketing environments. As the first validated and reliable marketing assessment tool, the FoodMATS represents a means to comprehensively track food marketing environments over time. With the forthcoming development of food marketing regulations in Canada or other countries, and the WHO’s call to restrict unhealthy food marketing in children’s settings [3], the FoodMATS may prove to be a fundamental ingredient in designing and monitoring regulatory interventions.

## Additional files

Additional file 1: Food & Beverage Marketing Checklist. (DOCX 112 kb)

Additional file 2: Components, definitions, and process of scoring data collected by the FoodMATS. (DOCX 17 kb)

Additional file 3: Nutrients and ingredients assessed in Canadian provincial nutrition guidelines for the recreation sector. (DOCX 19 kb)

Additional file 4: Categorization of food and beverage products recorded on FoodMATS and in concession sales data by harmonized criteria. (DOCX 24 kb)
