# Supplementary material for: Reliability and validity of a novel tool to comprehensively assess food and beverage marketing in recreational sport settings
Source: Int J Behav Nutr Phys Act. 2018 May 31;15:38. doi: 10.1186/s12966-018-0667-3 (PMC5977740; doi:10.1186/s12966-018-0667-3)
Supplement: Supplementary file 1 — Food & Beverage Marketing Checklist. (DOCX 112 kb) [file 12966_2018_667_MOESM1_ESM.docx]

**Additional file 1: Food & Beverage Marketing Checklist**

| **Community: ______________________________**  **Facility audited: ___________________________**  **🞎 Baseline:** Date**_________________** (mmm / dd / yyyy)  **🞎 Follow-up:** Date **_________________** (mmm / dd / yyyy)  This Assessment was completed by the following (check all that apply and fill in name):  □ **Provincial coordinator: _______________________**  □ **Research assistant:___________________________**  □ **Other (please specify): ________________________** |
| --- |

**Instructions**

The checklist is organized into the following sections:

1. **Parking Lot & Facility Grounds**
2. **Entrance, Reception & Hallways**
3. **Sport Areas**
   - There are 2 parts to Section 3:
4. All Sport Areas
5. Sport Areas with Food or Beverage Marketing
   - Assess all sport areas in the facility.
   - Each sport area with marketing will be filled out on individually with one page per Sport Area. If there are more than 5 sport areas with marketing, attach additional pages of Section 3. Remember to fill out the type of athletic area (and name if applicable) on the top of each page in Section 3B.
6. **Concession or Food Service Area**
   - This area includes commercial franchises.
   - There are 3 parts to Section 4:
7. Product Promotions
8. Pricing Promotions
9. Placement Promotions
   - Assess all concessions, food service areas, and commercial franchises in the facility.
   - There is space for two Concessions or Food Service Area in the checklist. Record marketing in all concessions. If there are more than 2 concessions, attach additional pages of Section 4A to 4C. If there is only 1, select “not applicable” beside “Concession 2” for all parts of Section 4.

You may want to consult a map of the facility (if available) to ensure you cover the entire facility.

1. Systematically walk through the facility documenting food and beverage marketing. Complete one area at a time, using the page designated for that section. For this checklist, **marketing** is defined as any commercial advertising, promotion, or messaging of food or beverage products/ brands/ food retailers (i.e. restaurant) that is intended to increase the “recognition, appeal and/or consumption” of such products/ brands/ retailer.
2. Within each section, review the suggested sites (i.e. outdoor facility sign) or marketing type (i.e. menu combos) and record the product/brand of all food or beverage products/ brands promoted. For example if a promotion says “Boston Pizza -visit us after the game”, record “Boston Pizza” as the **product/brand advertised**.

- Record each promotion separately. For example, if a vending machine has two promotions, one for Dasani Water and one for Vitamin Water, enter each promotion separately.
  - For each product promoted, record the product size and other product details (if possible).
- If no food or beverage marketing exists for that site or type, check the box indicating “No food/bev ads”.
- If that marketing site or type is does not exist at a facility, check the box indicating “Not applicable”.
- Add any additional promotions observed under “Other” and specify the location or type of marketing.

1. For each promotion you record, take a photo for verification and other purposes.
2. Where indicated, circle whether each promotion is **child-directed** or not. For example if a promotion says “Boston Pizza -visit us after the game” and has an image of a cartoon character, this would be considered child-directed.

Child-directed means that the promotion has evidence of animated or fictional characters, taste appeals, humour, action-adventure, fantasy, fun (shapes, colours), competitions, give-aways, cartoonish font, or uses a child actor to advertise a food or beverage product/brand that would appeal to children.

1. Where indicated, circle whether each promotion is **sports-related** or not. For example if a promotion says “Boston Pizza -visit us after the game”, because it refers to the “game” it would be considered sport-related.

Sports-related includes any reference to physical activity, exercise, sport, game, recreation, performance or competition.

1. Where indicated, circle the **size** of each promotion. Please note: the size of advertisements and promotions, defined as small, medium, and large, are different for indoor and outdoor promotions:

| Outdoor promotions: | Indoor promotions: |
| --- | --- |
| **small** < one letter size piece of paper (8.5 X 11 in)  **medium** 1-10 letter size sheets of paper together  **large** >10 pieces of paper together | **small** < one letter size piece of paper (8.5 X 11”)  **medium** 1-3 pieces of paper together  **large** >3 pieces of paper together |

- Please note: sizing for promotions on vending machines will always use indoor promotion sizing even if the machine is located outside.

1. If a promotion is entered into “Other”, add appropriate descriptive information (child-directed, size, etc.).
2. Once finished, review the entire tool before you leave the facility to ensure that everything is fully completed.
3. Enter data into provided spreadsheets. Number each promotion identified (site number_promotion number) in the spreadsheet and attach that number to the photo taken of that promotion.
4. Submit completed assessment form and photos (by email or downloaded into the data server) to Rachel Prowse for data checking.

**Section 1 - Parking Lot & Facility Grounds**

| **ID** | **Location** | **Product(s) or brand(s) advertised**  Record serving size of products | **Child-directed?** | **Sports-related?** | **Size of advertising^1^** |
| --- | --- | --- | --- | --- | --- |
| 1.1.1 | **1. Outdoor facility sign**  □ No ads  □ No food/bev ads  □ Not applicable |  | **Yes No** | **Yes No** | **S M L** |
| 1.1.2 |  |  | **Yes No** | **Yes No** | **S M L** |
| 1.2.1 | **2. Billboards**  □ No ads  □ No food/bev ads  □ Not applicable |  | **Yes No** | **Yes No** | **S M L** |
| 1.2.2 |  |  | **Yes No** | **Yes No** | **S M L** |
| 1.3.1 | **3. Temporary signs (i.e. sandwich boards)**  □ No ads  □ No food/bev ads  □ Not applicable |  | **Yes No** | **Yes No** | **S M L** |
| 1.3.2 |  |  | **Yes No** | **Yes No** | **S M L** |
| 1.3.3 |  |  | **Yes No** | **Yes No** | **S M L** |
| 1.4.1 | **4. Sides of building**  □ No ads  □ No food/bev ads |  | **Yes No** | **Yes No** | **S M L** |
| 1.4.2 |  |  | **Yes No** | **Yes No** | **S M L** |
| 1.4.3 |  |  | **Yes No** | **Yes No** | **S M L** |
| 1.5.1 | **5. Windows**  □ No ads  □ No food/bev ads  □ Not applicable |  | **Yes No** | **Yes No** | **S M L** |
| 1.5.2 |  |  | **Yes No** | **Yes No** | **S M L** |
| 1.5.3 |  |  | **Yes No** | **Yes No** | **S M L** |
| 1.6.1 | **6. Doors**  □ No ads  □ No food/bev ads |  | **Yes No** | **Yes No** | **S M L** |
| 1.6.2 |  |  | **Yes No** | **Yes No** | **S M L** |
| 1.6.3 |  |  | **Yes No** | **Yes No** | **S M L** |
| 1.7.1 | **7. Outdoor furniture**  **(i.e benches, tables, umbrellas)**  □ No ads  □ No food/bev ads  □ Not applicable | 1. □ Seasonal | **Yes No** | **Yes No** | **S M L** |
| 1.7.2 |  | 1. □ Seasonal | **Yes No** | **Yes No** | **S M L** |
| 1.7.3 |  | 1. □ Seasonal | **Yes No** | **Yes No** | **S M L** |
| 1.8.1 | **8. Vending machines^2^**  Total # of VM_______  □ No ads  □ No food/bev ads  □ Not applicable | 1. (VM#___) | **Yes No** | **Yes No** | **S M L** |
| 1.8.2 |  | 1. (VM#___) | **Yes No** | **Yes No** | **S M L** |
| 1.8.3 |  | 1. (VM#___) | **Yes No** | **Yes No** | **S M L** |
| 1.9.1 | **9. Other (specify)**  □ Not applicable |  | **Yes No** | **Yes No** | **S M L** |

^1^Size definitions: small < one letter size piece of paper (8.5 X 11 in); medium 1-10 letter size sheets of paper together; large >10 pieces of paper together

^2^Size definitions: small < one letter size piece of paper (8.5 X 11”); medium 1-3 pieces of paper together; large >3 pieces of paper together

**Section 2 - Entrance, Reception Area & Hallways**

| **ID** | **Location** | **Product(s) or brand(s) advertised**  Record serving size of products | **Child-directed?** | **Sports-related?** | **Size of advertising^2^** |
| --- | --- | --- | --- | --- | --- |
| 2.1.1 | **1. Facility pamphlets or brochures**  □ No ads  □ No food/bev ads  □ Not applicable |  | **Yes No** | **Yes No** | **S M L** |
| 2.1.2 |  |  | **Yes No** | **Yes No** | **S M L** |
| 2.2.1 | **2. Facility televisions**  □ No ads  □ No food/bev ads  □ Not applicable |  | **Yes No** | **Yes No** | **S M L** |
| 2.2.2 |  |  | **Yes No** | **Yes No** | **S M L** |
| 2.3.1 | **3. Welcome desk**  □ No ads  □ No food/bev ads  □ Not applicable |  | **Yes No** | **Yes No** | **S M L** |
| 2.3.2 |  |  | **Yes No** | **Yes No** | **S M L** |
| 2.3.3 |  |  | **Yes No** | **Yes No** | **S M L** |
| 2.4.1 | **4. Walls/ floors**  □ No ads  □ No food/bev ads |  | **Yes No** | **Yes No** | **S M L** |
| 2.4.2 |  |  | **Yes No** | **Yes No** | **S M L** |
| 2.4.3 |  |  | **Yes No** | **Yes No** | **S M L** |
| 2.5.1 | **5. Bathrooms**  □ No ads  □ No food/bev ads |  | **Yes No** | **Yes No** | **S M L** |
| 2.5.2 |  |  | **Yes No** | **Yes No** | **S M L** |
| 2.6.1 | **6. Vending machines**  Total # of VM_______  □ No ads  □ No food/bev ads  □ Not applicable | 1. (VM#___) | **Yes No** | **Yes No** | **S M L** |
| 2.6.2 |  | 1. (VM#___) | **Yes No** | **Yes No** | **S M L** |
| 2.6.3 |  | 1. (VM#___) | **Yes No** | **Yes No** | **S M L** |
| 2.6.4 |  | 1. (VM#___) | **Yes No** | **Yes No** | **S M L** |
| 2.6.5 |  | 1. (VM#___) | **Yes No** | **Yes No** | **S M L** |
| 2.6.6 |  | 1. (VM#___) | **Yes No** | **Yes No** | **S M L** |
| 2.6.7 |  | 1. (VM#___) | **Yes No** | **Yes No** | **S M L** |
| 2.6.8 |  | 1. (VM#___) | **Yes No** | **Yes No** | **S M L** |
| 2.6.9 |  | 1. (VM#___) | **Yes No** | **Yes No** | **S M L** |
| 2.6.10 |  | 1. (VM#___) | **Yes No** | **Yes No** | **S M L** |
| 2.6.11 |  | 1. (VM#___) | **Yes No** | **Yes No** | **S M L** |
| 2.6.12 |  | 1. (VM#___) | **Yes No** | **Yes No** | **S M L** |
| 2.7.1 | **7. Other (specify)**  □ Not applicable |  | **Yes No** | **Yes No** | **S M L** |

^2^Size definitions are as follows: small < one letter size piece of paper (8.5 X 11”); medium 1-3 pieces of paper together; large >3 pieces of paper together

**Section 3A - SPORT AREAS**

In the following table, record the type and number of sport areas present in the facility. Indicate the number of sport areas that have **no marketing,** the **number with non-food marketing ONLY**, and the number of sport areas **with food or beverage marketing.** The **Total number** should equal Number with **NO marketing** plus the number with **WITH non-food marketing ONLY** plus the number **WITH FOOD marketing.**  For sport areas with marketing, proceed to Section 3B.

| **Type of Indoor Sport Area** | **Total number** of number of sport areas | Number of sport areas with **NO marketing** | Number of sport areas **WITH non- food marketing**  **ONLY** | Number of sport areas **WITH FOOD marketing**  (Go to 3B) |
| --- | --- | --- | --- | --- |
| **Pool area** |  |  |  |  |
| **Playing field area** (indoor soccer field, etc.) |  |  |  |  |
| **Rink area** (including ice rinks that have been melted and used for another sport) |  |  |  |  |
| **Weight/cardio room area** |  |  |  |  |
| **Indoor track area** |  |  |  |  |
| **Cycling room area** |  |  |  |  |
| **Rock climbing space area** |  |  |  |  |
| **Single-use court (i.e. racket sports) area** |  |  |  |  |
| **Large multi-use gym (i.e. basketball) area** |  |  |  |  |
| **Small multi-use gym (i.e. yoga) area** |  |  |  |  |
| **Other area (specify)** |  |  |  |  |

Notes:

- Include indoor sports areas only.
- Record the number of “spaces” for these sports (not necessarily the number of fields or courts). For example, if there is one rink area for curling and the rink includes 5 sheets for 5 separate games, mark this as 1 area not 5. Similarly, if there is a collection of 6 tennis courts in 2 separate buildings, record this as 2 single use court areas, not 12 courts.
- For the areas WITH FOOD or beverage marketing, please fill out one page for each area and specify the type of athletic area in Section 3B. Attach more sport area pages if there are more than 5 athletic areas.

**Section 3B - SPORT AREA 1** (specify type of athletic area):

**Type** of athletic area (see Section 3A): ________________________________________________________

**Name** of athletic area (i.e. Ice Rink North or CIBC field) (if applicable): ________________________________

| **ID** | **Location** | **Product(s) or brand(s) advertised**  Record serving size of products | **Child-directed?** | | **Sports-related?** | | **Size of advertising^2^** | |  |
| --- | --- | --- | --- | --- | --- | --- | --- | --- | --- |
| 3-1. 1.1 | **1. Change/Locker rooms^3^**  □ No ads  □ No food/bev ads  □ Not applicable |  | **Yes No** | | **Yes No** | | **S M L** | |  |
| 3-1. 1.2 |  |  | **Yes No** | | **Yes No** | | **S M L** | |  |
| 3-1. 2.1 | **2. In playing area**  □ No ads  □ No food/bev ads  □ Not applicable |  | **Yes No** | | **Yes No** | | **S M L** | |  |
| 3-1. 2.2 |  |  | **Yes No** | | **Yes No** | | **S M L** | |  |
| 3-1. 2.3 |  |  | **Yes No** | | **Yes No** | | **S M L** | |  |
| 3-1. 2.4 |  |  | **Yes No** | | **Yes No** | | **S M L** | |  |
| 3-1. 2.5 |  |  | **Yes No** | | **Yes No** | | **S M L** | |  |
| 3-1. 3.1 | **3. On scoreboards**  □ No ads  □ No food/bev ads  □ Not applicable |  | **Yes No** | | **Yes No** | | **S M L** | |  |
| 3-1. 3.2 |  |  | **Yes No** | | **Yes No** | | **S M L** | |  |
| 3-1. 4.1 | **4. On clocks**  □ No ads  □ No food/bev ads  □ Not applicable |  | **Yes No** | | **Yes No** | | **S M L** | |  |
| 3-1. 4.2 |  |  | **Yes No** | | **Yes No** | | **S M L** | |  |
| 3-1. 5.1 | **5. In seating area**  □ No ads  □ No food/bev ads  □ Not applicable |  | **Yes No** | | **Yes No** | | **S M L** | |  |
| 3-1. 5.2 |  |  | **Yes No** | | **Yes No** | | **S M L** | |  |
| 3-1. 5.3 |  |  | **Yes No** | | **Yes No** | | **S M L** | |  |
| 3-1. 6.1 | **6. Vending machines in spectator area**  Total # of VM_______  □ No ads  □ No food/bev ads  □ Not applicable | 1. (VM#___) | | **Yes No** | | **Yes No** | | **S M L** | |
| 3-1. 6.2 |  | 1. (VM#___) | | **Yes No** | | **Yes No** | | **S M L** | |
| 3-1. 6.3 |  | 1. (VM#___) | | **Yes No** | | **Yes No** | | **S M L** | |
| 3-1. 7.1 | **7. Vending machines in athlete area**  Total # of VM_______  □ No ads  □ No food/bev ads  □ Not applicable | 1. (VM#___) | | **Yes No** | | **Yes No** | | **S M L** | |
| 3-1. 7.2 |  | 1. (VM#___) | | **Yes No** | | **Yes No** | | **S M L** | |
| 3-1. 7.3 |  | 1. (VM#___) | | **Yes No** | | **Yes No** | | **S M L** | |
| 3-1. 8.1 | **8. Other (specify)**  □ Not applicable |  | | **Yes No** | | **Yes No** | | **S M L** | |

^2^Sizes: small < one letter size piece of paper (8.5 X 11”); medium 1-3 pieces of paper together; large >3 pieces of paper together

^3^If there are multiple locker rooms, assess only the first three rooms encountered. If the change/locker rooms have already been accounted for, do not record the marketing here to prevent duplication.

**Section 3B - SPORT AREA 2** (specify type of athletic area):

Type of athletic area (see Section 3A): _________________________________________________________

Name of athletic area (i.e. Ice Rink North or CIBC field) (if applicable): ________________________________

| **ID** | **Location** | **Product(s) or brand(s) advertised**  Record serving size of products | **Child-directed?** | **Sports-related?** | **Size of advertising^2^** |
| --- | --- | --- | --- | --- | --- |
| 3-2. 1.1 | **1. Change/Locker rooms^3^**  □ No ads  □ No food/bev ads  □ Not applicable |  | **Yes No** | **Yes No** | **S M L** |
| 3-2. 1.2 |  |  | **Yes No** | **Yes No** | **S M L** |
| 3-2. 2.1 | **2. In playing area**  □ No ads  □ No food/bev ads  □ Not applicable |  | **Yes No** | **Yes No** | **S M L** |
| 3-2. 2.2 |  |  | **Yes No** | **Yes No** | **S M L** |
| 3-2. 2.3 |  |  | **Yes No** | **Yes No** | **S M L** |
| 3-2. 2.4 |  |  | **Yes No** | **Yes No** | **S M L** |
| 3-2. 2.5 |  |  | **Yes No** | **Yes No** | **S M L** |
| 3-2. 3.1 | **3. On scoreboards**  □ No ads  □ No food/bev ads  □ Not applicable |  | **Yes No** | **Yes No** | **S M L** |
| 3-2. 3.2 |  |  | **Yes No** | **Yes No** | **S M L** |
| 3-2. 4.1 | **4. On clocks**  □ No ads  □ No food/bev ads  □ Not applicable |  | **Yes No** | **Yes No** | **S M L** |
| 3-2. 4.2 |  |  | **Yes No** | **Yes No** | **S M L** |
| 3-2. 5.1 | **5. In seating area**  □ No ads  □ No food/bev ads  □ Not applicable |  | **Yes No** | **Yes No** | **S M L** |
| 3-2. 5.2 |  |  | **Yes No** | **Yes No** | **S M L** |
| 3-2. 5.3 |  |  | **Yes No** | **Yes No** | **S M L** |
| 3-2. 6.1 | **6. Vending machines in spectator area**  Total # of VM_______  □ No ads  □ No food/bev ads  □ Not applicable | 1. (VM#___) | **Yes No** | **Yes No** | **S M L** |
| 3-2. 6.2 |  | 1. (VM#___) | **Yes No** | **Yes No** | **S M L** |
| 3-2. 6.3 |  | 1. (VM#___) | **Yes No** | **Yes No** | **S M L** |
| 3-2. 7.1 | **7. Vending machines in athlete area**  Total # of VM_______  □ No ads  □ No food/bev ads  □ Not applicable | 1. (VM#___) | **Yes No** | **Yes No** | **S M L** |
| 3-2. 7.2 |  | 1. (VM#___) | **Yes No** | **Yes No** | **S M L** |
| 3-2. 7.3 |  | 1. (VM#___) | **Yes No** | **Yes No** | **S M L** |
| 3-2. 8.1 | **8. Other (specify)**  □ Not applicable |  | **Yes No** | **Yes No** | **S M L** |

^2^Sizes: small < one letter size piece of paper (8.5 X 11”); medium 1-3 pieces of paper together; large >3 pieces of paper together

^3^If there are multiple locker rooms, assess only the first three rooms encountered. If the change/locker rooms have already been accounted for, do not record the marketing here to prevent duplication.

**Section 3B - SPORT AREA 3** (specify type of athletic area):

Type of athletic area (see Section 3A): _________________________________________________________

Name of athletic area (i.e. Ice Rink North or CIBC field) (if applicable): ________________________________

| **ID** | **Location** | **Product(s) or brand(s) advertised**  Record serving size of products | **Child-directed?** | **Sports-related?** | **Size of advertising^2^** |
| --- | --- | --- | --- | --- | --- |
| 3-3. 1.1 | **1. Change/Locker rooms^3^**  □ No ads  □ No food/bev ads  □ Not applicable |  | **Yes No** | **Yes No** | **S M L** |
| 3-3. 1.2 |  |  | **Yes No** | **Yes No** | **S M L** |
| 3-3. 2.1 | **2. In playing area**  □ No ads  □ No food/bev ads  □ Not applicable |  | **Yes No** | **Yes No** | **S M L** |
| 3-3. 2.2 |  |  | **Yes No** | **Yes No** | **S M L** |
| 3-3. 2.3 |  |  | **Yes No** | **Yes No** | **S M L** |
| 3-3. 2.4 |  |  | **Yes No** | **Yes No** | **S M L** |
| 3-3. 2.5 |  |  | **Yes No** | **Yes No** | **S M L** |
| 3-3. 3.1 | **3. On scoreboards**  □ No ads  □ No food/bev ads  □ Not applicable |  | **Yes No** | **Yes No** | **S M L** |
| 3-3. 3.2 |  |  | **Yes No** | **Yes No** | **S M L** |
| 3-3. 4.1 | **4. On clocks**  □ No ads  □ No food/bev ads  □ Not applicable |  | **Yes No** | **Yes No** | **S M L** |
| 3-3. 4.2 |  |  | **Yes No** | **Yes No** | **S M L** |
| 3-3. 5.1 | **5. In seating area**  □ No ads  □ No food/bev ads  □ Not applicable |  | **Yes No** | **Yes No** | **S M L** |
| 3-3. 5.2 |  |  | **Yes No** | **Yes No** | **S M L** |
| 3-3. 5.3 |  |  | **Yes No** | **Yes No** | **S M L** |
| 3-3. 6.1 | **6. Vending machines in spectator area**  Total # of VM_______  □ No ads  □ No food/bev ads  □ Not applicable | 1. (VM#___) | **Yes No** | **Yes No** | **S M L** |
| 3-3. 6.2 |  | 1. (VM#___) | **Yes No** | **Yes No** | **S M L** |
| 3-3. 6.3 |  | 1. (VM#___) | **Yes No** | **Yes No** | **S M L** |
| 3-3. 7.1 | **7. Vending machines in athlete area**  Total # of VM_______  □ No ads  □ No food/bev ads  □ Not applicable | 1. (VM#___) | **Yes No** | **Yes No** | **S M L** |
| 3-3. 7.2 |  | 1. (VM#___) | **Yes No** | **Yes No** | **S M L** |
| 3-3. 7.3 |  | 1. (VM#___) | **Yes No** | **Yes No** | **S M L** |
| 3-3. 8.1 | **8. Other (specify)**  □ Not applicable |  | **Yes No** | **Yes No** | **S M L** |

^2^Sizes: small < one letter size piece of paper (8.5 X 11”); medium 1-3 pieces of paper together; large >3 pieces of paper together

^3^If there are multiple locker rooms, assess only the first three rooms encountered. If the change/locker rooms have already been accounted for, do not record the marketing here to prevent duplication.

**Section 3B - SPORT AREA 4** (specify type of athletic area):

Type of athletic area (see Section 3A): _________________________________________________________

Name of athletic area (i.e. Ice Rink North or CIBC field) (if applicable): ________________________________

| **ID** | **Location** | **Product(s) or brand(s) advertised**  Record serving size of products | **Child-directed?** | **Sports-related?** | **Size of advertising^2^** |
| --- | --- | --- | --- | --- | --- |
| 3-4. 1.1 | **1. Change/Locker rooms^3^**  □ No ads  □ No food/bev ads  □ Not applicable |  | **Yes No** | **Yes No** | **S M L** |
| 3-4. 1.2 |  |  | **Yes No** | **Yes No** | **S M L** |
| 3-4. 2.1 | **2. In playing area**  □ No ads  □ No food/bev ads  □ Not applicable |  | **Yes No** | **Yes No** | **S M L** |
| 3-4. 2.2 |  |  | **Yes No** | **Yes No** | **S M L** |
| 3-4. 2.3 |  |  | **Yes No** | **Yes No** | **S M L** |
| 3-4. 2.4 |  |  | **Yes No** | **Yes No** | **S M L** |
| 3-4. 2.5 |  |  | **Yes No** | **Yes No** | **S M L** |
| 3-4. 3.1 | **3. On scoreboards**  □ No ads  □ No food/bev ads  □ Not applicable |  | **Yes No** | **Yes No** | **S M L** |
| 3-4. 3.2 |  |  | **Yes No** | **Yes No** | **S M L** |
| 3-4. 4.1 | **4. On clocks**  □ No ads  □ No food/bev ads  □ Not applicable |  | **Yes No** | **Yes No** | **S M L** |
| 3-4. 4.2 |  |  | **Yes No** | **Yes No** | **S M L** |
| 3-4. 5.1 | **5. In seating area**  □ No ads  □ No food/bev ads  □ Not applicable |  | **Yes No** | **Yes No** | **S M L** |
| 3-4. 5.2 |  |  | **Yes No** | **Yes No** | **S M L** |
| 3-4. 5.3 |  |  | **Yes No** | **Yes No** | **S M L** |
| 3-4. 6.1 | **6. Vending machines in spectator area**  Total # of VM_______  □ No ads  □ No food/bev ads  □ Not applicable | 1. (VM#___) | **Yes No** | **Yes No** | **S M L** |
| 3-4. 6.2 |  | 1. (VM#___) | **Yes No** | **Yes No** | **S M L** |
| 3-4. 6.3 |  | 1. (VM#___) | **Yes No** | **Yes No** | **S M L** |
| 3-4. 7.1 | **7. Vending machines in athlete area**  Total # of VM_______  □ No ads  □ No food/bev ads  □ Not applicable | 1. (VM#___) | **Yes No** | **Yes No** | **S M L** |
| 3-4. 7.2 |  | 1. (VM#___) | **Yes No** | **Yes No** | **S M L** |
| 3-4. 7.3 |  | 1. (VM#___) | **Yes No** | **Yes No** | **S M L** |
| 3-4. 8.1 | **8. Other (specify)**  □ Not applicable |  | **Yes No** | **Yes No** | **S M L** |

^2^Sizes: small < one letter size piece of paper (8.5 X 11”); medium 1-3 pieces of paper together; large >3 pieces of paper together

^3^If there are multiple locker rooms, assess only the first three rooms encountered. If the change/locker rooms have already been accounted for, do not record the marketing here to prevent duplication.

**Section 3B - SPORT AREA 5** (specify type of athletic area):

Type of athletic area (see Section 3A): _________________________________________________________

Name of athletic area (i.e. Ice Rink North or CIBC field) (if applicable): ________________________________

| **ID** | **Location** | **Product(s) or brand(s) advertised**  Record serving size of products | **Child-directed?** | **Sports-related?** | **Size of advertising^2^** |
| --- | --- | --- | --- | --- | --- |
| 3-5. 1.1 | **1. Change/Locker rooms^3^**  □ No ads  □ No food/bev ads  □ Not applicable |  | **Yes No** | **Yes No** | **S M L** |
| 3-5. 1.2 |  |  | **Yes No** | **Yes No** | **S M L** |
| 3-5. 2.1 | **2. In playing area**  □ No ads  □ No food/bev ads  □ Not applicable |  | **Yes No** | **Yes No** | **S M L** |
| 3-5. 2.2 |  |  | **Yes No** | **Yes No** | **S M L** |
| 3-5. 2.3 |  |  | **Yes No** | **Yes No** | **S M L** |
| 3-5. 2.4 |  |  | **Yes No** | **Yes No** | **S M L** |
| 3-5. 2.5 |  |  | **Yes No** | **Yes No** | **S M L** |
| 3-5. 3.1 | **3. On scoreboards**  □ No ads  □ No food/bev ads  □ Not applicable |  | **Yes No** | **Yes No** | **S M L** |
| 3-5. 3.2 |  |  | **Yes No** | **Yes No** | **S M L** |
| 3-5. 4.1 | **4. On clocks**  □ No ads  □ No food/bev ads  □ Not applicable |  | **Yes No** | **Yes No** | **S M L** |
| 3-5. 4.2 |  |  | **Yes No** | **Yes No** | **S M L** |
| 3-5. 5.1 | **5. In seating area**  □ No ads  □ No food/bev ads  □ Not applicable |  | **Yes No** | **Yes No** | **S M L** |
| 3-5. 5.2 |  |  | **Yes No** | **Yes No** | **S M L** |
| 3-5. 5.3 |  |  | **Yes No** | **Yes No** | **S M L** |
| 3-5. 6.1 | **6. Vending machines in spectator area**  Total # of VM_______  □ No ads  □ No food/bev ads  □ Not applicable | 1. (VM#___) | **Yes No** | **Yes No** | **S M L** |
| 3-5. 6.2 |  | 1. (VM#___) | **Yes No** | **Yes No** | **S M L** |
| 3-5. 6.3 |  | 1. (VM#___) | **Yes No** | **Yes No** | **S M L** |
| 3-5. 7.1 | **7. Vending machines in athlete area**  Total # of VM_______  □ No ads  □ No food/bev ads  □ Not applicable | 1. (VM#___) | **Yes No** | **Yes No** | **S M L** |
| 3-5. 7.2 |  | 1. (VM#___) | **Yes No** | **Yes No** | **S M L** |
| 3-5. 7.3 |  | 1. (VM#___) | **Yes No** | **Yes No** | **S M L** |
| 3-5. 8.1 | **8. Other (specify)**  □ Not applicable |  | **Yes No** | **Yes No** | **S M L** |

^2^Sizes: small < one letter size piece of paper (8.5 X 11”); medium 1-3 pieces of paper together; large >3 pieces of paper together

^3^If there are multiple locker rooms, assess only the first three rooms encountered. If the change/locker rooms have already been accounted for, do not record the marketing here to prevent duplication.

**Section 4 - Concession or Food Service Areas** A. Menu Item Promotions

Name of Concession/Franchise: ________________________________

| **4-1** | **CONCESSION 1** | | | | | | |
| --- | --- | --- | --- | --- | --- | --- | --- |
| **ID** | | **Marketing Type** | **Product(s) or brand(s) advertised**  Record serving size of products | **Child-directed?** | **Sports-related?** | **Size of advertising^2^** | |
| 4-1a .1.1 | | **1. Menu signs/ messages/ etc. that promote specific menu items^4^**  □ Not applicable  *Example: Menu says: “Try our new hearty tomato soup”*  Does not include listing of menu items in general |  | **Yes No** | **Yes No** | **S M L** | |
| 4-1a .1.2 | |  |  | **Yes No** | **Yes No** | **S M L** | |
| 4-1a .1.3 | |  |  | **Yes No** | **Yes No** | **S M L** | |
| 4-1a .1.4 | |  |  | **Yes No** | **Yes No** | **S M L** | |
| 4-1a .1.5 | |  |  | **Yes No** | **Yes No** | **S M L** | |
| 4-1a .1.6 | |  |  | **Yes No** | **Yes No** | **S M L** | |
| 4-1a .1.7 | |  |  | **Yes No** | **Yes No** | **S M L** | |
| 4-1a .2.1 | | **2. Menu signs/ messages/ etc. that promote children’s menu items**  □ No food/bev ads  □ Not applicable |  | **Yes No** | **Yes No** | **S M L** | |
| 4-1a .2.2 | |  |  | **Yes No** | **Yes No** | **S M L** | |
| 4-1a .2.3 | |  |  | **Yes No** | **Yes No** | **S M L** | |
| 4-1a .2.4 | |  |  | **Yes No** | **Yes No** | **S M L** | |
| 4-1a .3.1 | | **3. Healthy items identified on menu**  □ Not applicable | Describe how items are identified and what items are identified as healthy: | | | | |
| 4-1a .4.1 | | **4. Other signs/ table tents/ displays^5^ that promote specific menu items**  □ Not applicable  *Example: Sign at entrance of concession says: “Cold and refreshing fruit smoothies”* |  | **Yes No** | **Yes No** | | **S M L** |
| 4-1a .4.2 | |  |  | **Yes No** | **Yes No** | | **S M L** |
| 4-1a .4.3 | |  |  | **Yes No** | **Yes No** | | **S M L** |
| 4-1a .4.4 | |  |  | **Yes No** | **Yes No** | | **S M L** |
| 4-1a .4.5 | |  |  | **Yes No** | **Yes No** | | **S M L** |
| 4-1a .4.6 | |  |  | **Yes No** | **Yes No** | | **S M L** |
| 4-1a .7.1 | | **5. Vending machines**  Total # of VM_______  □ No ads  □ No food/bev ads  □ Not applicable | 1. (VM#___) | **Yes No** | **Yes No** | | **S M L** |
| 4-1a .7.2 | |  | 1. (VM#___) | **Yes No** | **Yes No** | | **S M L** |
| 4-1a .7.3 | |  | 1. (VM#___) | **Yes No** | **Yes No** | | **S M L** |
| 4-1a .8.1 | | **8. Other (specify)**  □ Not applicable |  | **Yes No** | **Yes No** | | **S M L** |

^3^Sizes: small < one letter size piece of paper (8.5 X 11”); medium 1-3 pieces of paper together; large >3 pieces of paper together ; ^4^“Menu signs/messages that promote specific menu items” includes any signs on or around the menu board that highlights a particular menu item or brand. It does not include menus in general. Other signage (i.e. on walls) for particular products should be captured under “Other signs/table tents/displays that promote specific menu items”. ^5^ “Other signs/ table tents/ displays that promote specific menu items” includes other signage or displays in the concession that promotes a particular menu item or brand.

**Section 4 - Concession or Food Service Areas** B. Pricing Promotions

| **4-1** | **CONCESSION 1** | | | | | | |
| --- | --- | --- | --- | --- | --- | --- | --- |
| **ID** | **Marketing Type** | **Product(s) or brand(s) advertised**  Record serving size of products | | **Child-directed?** | | **Sports-related?** | **Size of advertising^2^** |
| 4-1b .1.1 | **1. Supersize**  □ Not applicable |  | | **Yes No** | | **Yes No** | **S M L** |
| 4-1a .2.1 | **2. All-you-can-eat or “unlimited trips”**  □ Not applicable |  | | **Yes No** | | **Yes No** | **S M L** |
| 4-1a .2.2 |  |  | | **Yes No** | | **Yes No** | **S M L** |
| 4-1a .3.1 | **3. Free refills**  □ Not applicable |  | | **Yes No** | | **Yes No** | **S M L** |
| 4-1a .3.1 |  |  | | **Yes No** | | **Yes No** | **S M L** |
| 4-1a .4.1 | **4. Loyalty programs/ cards** □ Not applicable |  | | **Yes No** | | **Yes No** | **S M L** |
|  |  | | | | | | |
|  | **Marketing Type** | | **Product(s) or brand(s) advertised**  Record serving size of products | | | | |
| 4-1a .5.1 | **5. Sum of individual items compared to combo meals**  □ Not applicable | | **Individual items:** | | **Combo:** | | |
|  |  |  | **$** | | **$** | | |
| 4-1a .6.1 | **6. Smaller portion compared to regular portion^6^**  □ Not applicable | | **Small portion:** | | **Regular portion:** | | |
|  |  |  | **$** | | **$** | | |
| 4-1a .7.1 | **7. Healthy entrees^7^ compared to regular ones in the concession**  □ Not applicable | | **Healthy entrée:** | | **Regular entrée:** | | |
|  |  |  | **$** | | **$** | | |
| 4-1a .8.1 | **8. Healthy main dish salads^8^ compared to regular ones in the concession**  □ Not applicable | | **Healthy salad:** | | **Regular salad:** | | |
|  |  |  | **$** | | **$** | | |
| 4-1a .9.1 | **9. Healthy beverages^9^ compared to regular ones in the concession**  □ Not applicable | | **Water/milk/juice:** | | **Sweetened water:** (pop, vitamin water, Gatorade) | | |
|  |  |  | **$** | | **$** | | |
| 4-1a .10.1 | **10. Healthy beverages^9^ compared to regular ones in a vending machine** (closest to concession)  □ Not applicable VM#___ | | **Water:** | | **Sweetened water:** (pop, vitamin water, Gatorade) | | |
|  |  |  | **$** | | **$** | | |
| 4-1a .11.1 | **11. Healthy snacks^10^ compared to regular ones in the concession**  □ Not applicable | | **Healthy snack:** (fruit/vegetable) | | **Regular snack:** (cookie/chips/choco) | | |
|  |  |  | **$** | | **$** | | |
| 4-1a .12.1 | **12. Other (specify)**  □ Not applicable | |  | | | | |

^2^Sizes: small < one letter size piece of paper (8.5 X 11”); medium 1-3 pieces of paper together; large >3 pieces of paper together

^6^The price should be relative (i.e. a half portion should cost half as much as the full portion)

^7^A healthy main dish/ entrée is defined as per our NEMS plus definition.

^8^A healthy main dish salad is defined as per our NEMS plus definition.

^9^Choose beverages comparable in size. Use water (or milk, or juice) for the healthy beverage, and pick a sugar beverage made from water (or milk) as the regular beverage, such as pop, Vitamin Water, or Gatorade (or chocolate milk).

^10^ Choose and specify one healthy compare and one regular snack to compare using the examples provided, provided adequate details.

**Section 4 - Concession or Food Service Areas** C. Placement Promotions

| **4-1** | **CONCESSION 1** | | | | | | | | | | |
| --- | --- | --- | --- | --- | --- | --- | --- | --- | --- | --- | --- |
| **ID** | **Location** | **Product(s) or brand(s) advertised**  Record serving size of products | | | **Child-directed?** | | | **Sports-related?** | | | **Size of advertising^2^** |
| 4-1c .1.1 | **1. At checkout**  Include non-packaged products and advertisements located at the checkout^11^  **Record any packaged products (that would be sold in vending machines) in the concession audit.**  □ No ads  □ No food/bev ads  □ Not applicable |  | | **Yes No N/A** | | | **Yes No N/A** | | | **S M L N/A** | |
| 4-1c .1.2 |  |  | | **Yes No N/A** | | | **Yes No N/A** | | | **S M L N/A** | |
| 4-1c .1.3 |  |  | | **Yes No N/A** | | | **Yes No N/A** | | | **S M L N/A** | |
| 4-1c .1.4 |  |  | | **Yes No N/A** | | | **Yes No N/A** | | | **S M L N/A** | |
| 4-1c .1.5 |  |  | | **Yes No N/A** | | | **Yes No N/A** | | | **S M L N/A** | |
| 4-1c .1.6 |  |  | | **Yes No N/A** | | | **Yes No N/A** | | | **S M L N/A** | |
| 4-1c .1.7 |  |  | | **Yes No N/A** | | | **Yes No N/A** | | | **S M L N/A** | |
| 4-1c .1.8 |  |  | | **Yes No N/A** | | | **Yes No N/A** | | | **S M L N/A** | |
| 4-1c .1.9 |  |  | | **Yes No N/A** | | | **Yes No N/A** | | | **S M L N/A** | |
| 4-1c .1.10 |  |  | | **Yes No N/A** | | | **Yes No N/A** | | | **S M L N/A** | |
| 4-1c .2.1 | **2. Other (specify)**  □ Not applicable |  | **Yes No N/A** | | | **Yes No N/A** | | | **S M L N/A** | | |

^2^Size: small < one letter size piece of paper (8.5 X 11”); medium 1-3 pieces of paper together; large >3 pieces of paper together

^11^Checkout is defined as the space close to you (within reach) when paying for your order (what you see when you are buying your food or beverage).

**Section 4 - Concession or Food Service Areas** A. Menu Item Promotions

Name of Concession/Franchise: ________________________________

| **4-2** | **CONCESSION 2** □ Not applicable | | | | |
| --- | --- | --- | --- | --- | --- |
| **ID** | **Marketing Type** | **Product(s) or brand(s) advertised** | **Child-directed?** | **Sports-related?** | **Size of advertising^2^** |
| 4-2a .1.1 | **1. Menu signs/ messages/ etc. that promote specific menu items^4^**  □ Not applicable  *Example: Menu says: “Try our new hearty tomato soup”*  Does not include listing of menu items in general |  | **Yes No** | **Yes No** | **S M L** |
| 4-2a .1.2 |  |  | **Yes No** | **Yes No** | **S M L** |
| 4-2a .1.3 |  |  | **Yes No** | **Yes No** | **S M L** |
| 4-2a .1.4 |  |  | **Yes No** | **Yes No** | **S M L** |
| 4-2a .1.5 |  |  | **Yes No** | **Yes No** | **S M L** |
| 4-2a .1.6 |  |  | **Yes No** | **Yes No** | **S M L** |
| 4-2a .1.7 |  |  | **Yes No** | **Yes No** | **S M L** |
| 4-2a .2.1 | **2. Menu signs/ messages/ etc. that promote children’s menu items**  □ No food/bev ads  □ Not applicable |  | **Yes No** | **Yes No** | **S M L** |
| 4-2a .2.2 |  |  | **Yes No** | **Yes No** | **S M L** |
| 4-2a .2.3 |  |  | **Yes No** | **Yes No** | **S M L** |
| 4-2a .2.4 |  |  | **Yes No** | **Yes No** | **S M L** |
| 4-2a .3.1 | **3. Healthy items identified on menu**  □ Not applicable | Describe how items are identified and what items are identified as healthy: | | | |
| 4-2a .4.1 | **4. Other signs/ table tents/ displays^5^ that promote specific menu items**  □ Not applicable  *Example: Sign at entrance of concession says: “Cold and refreshing fruit smoothies”* |  | **Yes No** | **Yes No** | **S M L** |
| 4-2a .4.2 |  |  | **Yes No** | **Yes No** | **S M L** |
| 4-2a .4.3 |  |  | **Yes No** | **Yes No** | **S M L** |
| 4-2a .4.4 |  |  | **Yes No** | **Yes No** | **S M L** |
| 4-2a .4.5 |  |  | **Yes No** | **Yes No** | **S M L** |
| 4-2a .4.6 |  |  | **Yes No** | **Yes No** | **S M L** |
| 4-2a .5.1 | **5. Vending machines**  Total # of VM_______  □ No ads  □ No food/bev ads  □ Not applicable | 1. (VM#___)^2^ | **Yes No** | **Yes No** | **S M L** |
| 4-2a .5.2 |  | 1. (VM#___)^2^ | **Yes No** | **Yes No** | **S M L** |
| 4-2a .5.3 |  | 1. (VM#___)^2^ | **Yes No** | **Yes No** | **S M L** |
| 4-1a .8.1 | **8. Other (specify)**  □ Not applicable |  | **Yes No** | **Yes No** | **S M L** |

^3^Sizes: small < one letter size piece of paper (8.5 X 11”); medium 1-3 pieces of paper together; large >3 pieces of paper together ; ^4^“Menu signs/messages that promote specific menu items” includes any signs on or around the menu board that highlights a particular menu item or brand. It does not include menus in general. Other signage (i.e. on walls) for particular products should be captured under “Other signs/table tents/displays that promote specific menu items”. ^5^ “Other signs/ table tents/ displays that promote specific menu items” includes other signage or displays in the concession that promotes a particular menu item or brand.

**Section 4 - Concession or Food Service Areas** B. Pricing Promotions

| **4-2** | **CONCESSION 2** □ Not applicable | | | | | | |
| --- | --- | --- | --- | --- | --- | --- | --- |
| **ID** | **Marketing Type** | **Product(s) or brand(s) advertised** | | **Child-directed?** | | **Sports-related?** | **Size of advertising^2^** |
| 4-2b .1.1 | **1. Supersize**  □ Not applicable |  | | **Yes No** | | **Yes No** | **S M L** |
| 4-2a .2.1 | **2. All-you-can-eat or “unlimited trips”**  □ Not applicable |  | | **Yes No** | | **Yes No** | **S M L** |
| 4-2a .2.2 |  |  | | **Yes No** | | **Yes No** | **S M L** |
| 4-2a .3.1 | **3. Free refills**  □ Not applicable |  | | **Yes No** | | **Yes No** | **S M L** |
| 4-2a .3.1 |  |  | | **Yes No** | | **Yes No** | **S M L** |
| 4-2a .4.1 | **4. Loyalty programs/ cards** □ Not applicable |  | | **Yes No** | | **Yes No** | **S M L** |
|  |  | | | | | | |
| **ID** | **Marketing Type** | | **Product(s) or brand(s) advertised**  Record serving size of products | | | | |
| 4-2a .5.1 | **5. Sum of individual items compared to combo meals**  □ Not applicable | | **Individual items:** | | **Combo:** | | |
|  |  |  | **$** | | **$** | | |
| 4-2a .6.1 | **6. Smaller portion compared to regular portion^6^**  □ Not applicable | | **Small portion** (record size)**:** | | **Regular portion** (record size**:** | | |
|  |  |  | **$** | | **$** | | |
| 4-2a .7.1 | **7. Healthy entrees^9^ compared to regular ones in the concession**  □ Not applicable | | **Healthy entrée:** | | **Regular entrée:** | | |
|  |  |  | **$** | | **$** | | |
| 4-2a .8.1 | **8. Healthy main dish salads^8^ compared to regular ones in the concession**  □ Not applicable | | **Healthy salad:** | | **Regular salad:** | | |
|  |  |  | **$** | | **$** | | |
| 4-2a .9.1 | **9. Healthy beverages^9^ compared to regular ones in the concession**  □ Not applicable | | **Water:** | | **Sweetened water:** (pop, vitamin water, Gatorade) | | |
|  |  |  | **$** | | **$** | | |
| 4-2a .10.1 | **10. Healthy beverages^9^ compared to regular ones in a vending machine** (closest to concession)  □ Not applicable VM#___ | | **Water:** | | **Sweetened water:** (pop, vitamin water, Gatorade) | | |
|  |  |  | **$** | | **$** | | |
| 4-2a .11.1 | **11. Healthy snacks^10^ compared to regular ones in the concession**  □ Not applicable | | **Healthy snack:** (fruit/vegetable) | | **Regular snack:** (cookie/chips/choco) | | |
|  |  |  | **$** | | **$** | | |
| 4-2a .12.1 | **12. Other (specify)**  □ Not applicable | |  | | | | |

^2^Sizes: small < one letter size piece of paper (8.5 X 11”); medium 1-3 pieces of paper together; large >3 pieces of paper together

^6^The price should be relative (i.e. a half portion should cost half as much as the full portion)

^7^A healthy main dish/ entrée is defined as per our NEMS plus definition.

^8^A healthy main dish salad is defined as per our NEMS plus definition.

^9^Choose beverages comparable in size. Use water (or milk, or juice) for the healthy beverage, and pick a sugar beverage made from water (or milk) as the regular beverage, such as pop, Vitamin Water, or Gatorade (or chocolate milk).

^10^ Choose and specify one healthy compare and one regular snack to compare using the examples provided, provided adequate details.

**Section 4 - Concession or Food Service Areas** C. Placement Promotions

| **4-2** | **CONCESSION 2** □ Not applicable | | | | |
| --- | --- | --- | --- | --- | --- |
| **ID** | **Location** | **Product(s) or brand(s) advertised**  **Record servings size of products** | **Child-directed?** | **Sports-related?** | **Size of advertising^2^** |
| 4-2c .1.1 | **1. At checkout**  Include non-packaged products and advertisements located at the checkout^11^  **Record any packaged products (that would be sold in vending machines) in the concession audit.**  □ No ads  □ No food/bev ads  □ Not applicable |  | **Yes No N/A** | **Yes No N/A** | **S M L N/A** |
| 4-2c .1.2 |  |  | **Yes No N/A** | **Yes No N/A** | **S M L N/A** |
| 4-2c .1.3 |  |  | **Yes No N/A** | **Yes No N/A** | **S M L N/A** |
| 4-2c .1.4 |  |  | **Yes No N/A** | **Yes No N/A** | **S M L N/A** |
| 4-2c .1.5 |  |  | **Yes No N/A** | **Yes No N/A** | **S M L N/A** |
| 4-2c .1.6 |  |  | **Yes No N/A** | **Yes No N/A** | **S M L N/A** |
| 4-2c .1.7 |  |  | **Yes No N/A** | **Yes No N/A** | **S M L N/A** |
| 4-2c .1.8 |  |  | **Yes No N/A** | **Yes No N/A** | **S M L N/A** |
| 4-2c .1.9 |  |  | **Yes No N/A** | **Yes No N/A** | **S M L N/A** |
| 4-2c .1.10 |  |  | **Yes No N/A** | **Yes No N/A** | **S M L N/A** |
| 4-2c .2.1 | **2. Other (specify)**  □ Not applicable |  | **Yes No N/A** | **Yes No N/A** | **S M L N/A** |

^2^Size: small < one letter size piece of paper (8.5 X 11”); medium 1-3 pieces of paper together; large >3 pieces of paper together

^11^Checkout is defined as the space close to you (within reach) when paying for your order (what you see when you are buying your food or beverage).

**Review entire assessment form prior to leaving the facility to ensure it is fully completed.**
