# Supplementary material for: Reliability and validity of a novel tool to comprehensively assess food and beverage marketing in recreational sport settings
Source: Int J Behav Nutr Phys Act. 2018 May 31;15:38. doi: 10.1186/s12966-018-0667-3 (PMC5977740; doi:10.1186/s12966-018-0667-3)
Supplement: Supplementary file 2 — Components, definitions, and process of scoring data collected by the FoodMATS. (DOCX 17 kb) [file 12966_2018_667_MOESM2_ESM.docx]

Additional file 2: Components, definitions, and process of scoring data collected by the FoodMATS

| Component | Exposure | | Power | | | | | | |
| --- | --- | --- | --- | --- | --- | --- | --- | --- | --- |
| Indicator | Frequency | Repetition | Content | | | Design^2^ | | Execution^2^ | |
| Definition | Any commercial advertising, promotion, or messaging of food or beverage products/ brands/ food retailers (i.e. restaurant) that is intended to increase the “recognition, appeal and/or consumption” of such products/ brands [26] (p.9)  Excludes product packaging. | A product, brand, or food retailer that is marketing ≥ 3 times within 1 facility. | Product/Brands:  “Most Healthy”= unprocessed food/beverages with no added fat, sugar or salt; “Less Healthy”= some added fat, sugar, or salt; “Least Healthy”= processed energy-dense, nutrient-poor items with high levels of fat, sugar, or salt. | | Retailers:  “Most Healthy”= sandwich outlets, smoothie outlets, grocery stores, farmers’ markets, and salad bars; “Less Healthy”=sit-down restaurants, cafeterias, coffee outlets, prepared grocery stores, and supplement stores; “Least Healthy”= pizza, burger, taco, fried chicken, Asian, and ice cream outlets, and pubs/lounges/alcohol stores. | Evidence of animated or fictional characters, taste appeals, humour, action-adventure, fantasy, fun (shapes, colours), competitions, give-aways, [2] cartoonish font [22], or uses a child actor^1^ to advertise a food or beverage product/brand that would appeal to children. | Any reference to physical activity, exercise, sport, game, recreation, performance or competition. (A design feature relevant to sport settings) | Outdoor [19]:  small < one letter size piece of paper (8.5 X 11 in)  medium 1-10 letter size sheets of paper together  large >10 pieces of paper together | Indoor [16]:  small < one letter size piece of paper (8.5 X 11”)  medium 1-3 pieces of paper together  large >3 pieces of paper together |
| Every marketing instance identified was ranked on each indicator using the definitions: | | | | | | | | | |
| Rankings | One *instance*=1 | One *repeated* product, brand or retailer=1 | *Ranked* as “Least Healthy”=1; “Less Healthy”=0.5; “Most Healthy”=0 | | | *Ranked* as present=1; or absent=0 | *Ranked* as present=1; or absent=0 | *Ranked* as large=1; medium=0.5; small=0 | |
| Rankings for each marketing instance were scored within each food, sport, other area: | | | | | | | | | |
| Indicator Scores | FREQ = ∑*instances** 0.2 pts | REP = ∑*repeated**1 pt | | UNHE=∑*rankings* / FREQ * 5 pts | | CHIL = ∑*rankings* / FREQ * 5 pts | SPOR = ∑*rankings* / FREQ * 5 pts | SIZE = ∑*rankings* / FREQ * 5 pts | |
| For each area (food, sport, other), a FoodMATS score was calculated. If there was more than one sports area or food area within one facility, each area was scored individually and then summed for the complete Sport or Food Area score. | | | | | | | | | |
| Area Scores | FOODMATS_Area_ = FREQ + (FREQ*POW) where POW = UNHE + CHIL + SPOR + SIZE | | | | | | | | |
| For the entire site, a total FoodMATS score was calculated by summing all Area scores and adding a repetition factor to reflect the number of repeated products, brands, retailers marketed per site. | | | | | | | | | |
| Facility Scores | FOODMATS_Facility_ = FOODMATS_Sports_ + FOODMATS_Food_ + FOODMATS_Other_ + REP | | | | | | | | |

^1^added post pilot after this technique was identified; ^2^excludes pricing and select place marketing instance
