# Supplementary material for: Reliability and validity of a novel tool to comprehensively assess food and beverage marketing in recreational sport settings
Source: Int J Behav Nutr Phys Act. 2018 May 31;15:38. doi: 10.1186/s12966-018-0667-3 (PMC5977740; doi:10.1186/s12966-018-0667-3)
Supplement: Supplementary file 3 — Nutrients and ingredients assessed in Canadian provincial nutrition guidelines for the recreation sector. (DOCX 19 kb) [file 12966_2018_667_MOESM3_ESM.docx]

Additional file 3: Nutrient and ingredients assessed in Canadian provincial nutrition guidelines for the recreation sector

| Province | Guidelines | Year | Raking Categories | Reference amount | Nutrients Assessed | | | | | | | | | | | |
| --- | --- | --- | --- | --- | --- | --- | --- | --- | --- | --- | --- | --- | --- | --- | --- | --- |
|  |  |  |  |  | Energy (kcal) | Fat (g) | SF^1^ (g) | TF^2^ (g) | Na^3^ (mg) | Sugar (g) | Fibre (g) | Protein (g) | Ca^4^ (%DV) | Fe^5^ (%DV) | Vit D^6^ (%DV) | Ingredients assessed |
| Alberta | Alberta Nutrition Guidelines for Children and Youth for childcare, school, and recreation/ community centres | Introduced: 2008  Updated: 2010 | 2008-present:  (1) “Choose Most Often”  (2) “Choose Sometimes”  (3) “Choose Least Often” | Serving size based on product type | ✓^7^ | ✓ | ✓ | ✓ | ✓ | ✓ | ✓ | ✓ | ✓ | ✓ | ✓ | - Added fibre - Added fat - Added sugar - Artificial sweeteners - Caffeine |
| British Columba | Healthier Choices in Vending Machines in B.C. Public Buildings | Introduced: 2006  Updated 2014 | 2006-2014:  (1) “Choose Most Often”  (2) “Choose Sometimes”  (3) “Choose Least Often”  (4) “Not Recommended”  2014-present:  (1) “Sell Most”  (2) “Sell Sometimes”  (3) “Do Not Sell” | Package size | ✓ | ✓ | ✓ | ✓ | ✓ | ✓ | 🗶^8^ | ✓ | ✓ | 🗶 | ✓ | - Whole grain - Added sugar - Artificial sweeteners - Caffeine - Other botanical ingredients |
| Nova Scotia | Guidelines for Healthy Eating in Recreation and Sport Settings | Introduced: 2016 | 2016-present:  (1) “Maximum”  (2) “Moderate”  (3) “Minimum” | Serving size based on product type | ✓ | ✓ | ✓ | ✓ | ✓ | ✓ | 🗶 | ✓ | 🗶 | 🗶 | 🗶 | - Added fibre - Added fat - Added sugar - Added sodium - Artificial sweeteners - Caffeine |

^1^SF=saturated fat ^2^TF= trans fat ^3^Na=sodium ^4^Ca=calcium ^5^Fe=iron ^6^Vit D=vitamin D ^7^✓=included nutrient in profiling system ^8^🗶=excluded nutrient in profiling system
