# Supplementary material for: Reliability and validity of a novel tool to comprehensively assess food and beverage marketing in recreational sport settings
Source: Int J Behav Nutr Phys Act. 2018 May 31;15:38. doi: 10.1186/s12966-018-0667-3 (PMC5977740; doi:10.1186/s12966-018-0667-3)
Supplement: Supplementary file 4 — Categorization of food and beverage products recorded on FoodMATS and in concession sales data by harmonized criteria. (DOCX 24 kb) [file 12966_2018_667_MOESM4_ESM.docx]

Additional file 4: Categorization of food and beverage products recorded on FoodMATS and in concession sales data by harmonized criteria

| Food Type | Harmonized Categories | | | | | | |
| --- | --- | --- | --- | --- | --- | --- | --- |
|  | Most Healthy  *With no added fat, sugar, or salt* | | | Less Healthy  *With 1 added fat, sugar, or salt; OR low levels of 2 added fat, sugar, or salt* | | | Least Healthy  *With 2 added fat, sugar, or salt; OR high levels of 1 added fat, sugar, or salt* |
| Vegetables & Fruit | Fresh, frozen, canned fruit/vegetables  Fruit/vegetables with dip/dressing  Dried fruit  Fruit sauce | | | Smoothies  100% fruit or vegetable juice  Baked vegetable chips | | | Regular vegetable chips  Deep fried vegetables  Pickled vegetables |
| Grain Products | *Must be whole grain.*  Whole grain rice, pasta, bread, pitas, wraps  Hot cereal | | | *May be whole grain or refined grain.*  Granola bars  Non-whole grain rice, pasta, bread, pitas, wraps  Baked goods (e.g. muffins)  Rice cakes  Pretzels (hard) | | | *Refined grain products.*  Cold cereal  Pancakes  Cookies  Dessert-like baked goods (e.g. brownie)  Crackers  Pretzels (soft) |
| Milk & Alternatives | Plain skim, 1%, 2% milk  Plain skim, 1%, 2% yogurt, kefir, soygurt  Low-fat (<20% M.F.) cheese | | | Flavoured skim, 1%, 2% or higher fat yogurt, kefir, soygurt  Regular fat cheese (>20% M.F.) | | | Sweetened milk  Processed cheese products |
| Meat & Alternatives | Fresh, frozen lean meat, poultry, fish that was baked, broiled, grilled  Eggs  Legumes  Nut/seeds, nut butters | | | Hummus  Mayonnaise-based egg, tuna salads  Salted/sugared nut, seeds, nut butters | | | Breaded and/or deep-fried meat, poultry, fish  Processed meat (deli, bacon, sausage, jerky) |
| Mixed dish with no protein source^[[1]](#footnote-1)^ | - Each major ingredient of the dish assessed according to their food type. Closest ranking to the average of all ingredients is selected as the rank for that mixed product. If the average was exactly in the middle of “Most Healthy” and “Less Healthy” it was increased to “Most Healthy”. If the average was exactly in the middle of “Least Healthy” and “Less Healthy” it was lowered to “Least Healthy”. - If the mixed dish included an undefined beverage (i.e. “drink”), the ranking of the mixed dish alone served as the ranking of the combo (mixed dish + drink). If the mixed dish included a defined beverage (i.e. “juice”), the beverage was included as a major ingredient of the mixed dish and was included in the calculation of the average ranking. | | | | | | |
| Mixed dish with a protein source^3^  (entrees) | - Entire dish assessed on evidence of 4 qualities: (1) whole grain^[[2]](#footnote-2)^ (if applicable), (2) quality protein^[[3]](#footnote-3)^, (3) vegetable serving^[[4]](#footnote-4)^, (4) no added fat^[[5]](#footnote-5)^. - A priori simplifying assumptions were necessary to classify mixed dishes with protein (see below). Additional information was obtained from menu analyses for concession audits when available to inform ranking. - Rankings for combos of mixed dishes with protein and beverages were calculated by averaging the ranking of the mixed dish with protein and the ranking of the beverage. If the mixed dish with protein included an undefined beverage (i.e. “drink”), the ranking of the mixed dish with protein alone served as the ranking of the combo. - Averages that fall exactly between two categories were treated the same as above (see “Mixed dish with no protein”). | | | | | | |
|  | Evidence of all 4/4 qualities | | Evidence of all 2-3/4 qualities | | | Evidence of all 0-1/4 qualities | |
| Kids’ Meals | - Assessed on evidence of 3 qualities: (1) whole grain^17^ (if applicable), (2) quality protein^18^, (3) healthy side^[[6]](#footnote-6)^. Kids’ meals were automatically ranked as “Least Healthy” if they contained red meat or had cheese as a major ingredient. | | | | | | |
|  | Evidence of all 3/3 qualities | Evidence of 2/3 qualities | | | Evidence of 0-1/3 qualities, OR contains red meat or cheese as their major ingredient^[[7]](#footnote-7)^. | | |
| Condiments | None. | Sauces that provide some nutritional value and/or have low levels of fat, sugar, or salt:  Salsa (homemade)  Low-fat salad dressing | | | Sauces that provide no nutritional value and/or have high levels of fat, sugar, or salt:  Sour cream  Cream cheese  Gravy | | |
| Other foods | None. | None. | | | Energy-dense, nutrient poor foods:  Chocolate  Candy  Ice cream/frozen desserts  Baked desserts (e.g. pie) | | |
| Other beverages | Beverages with no added sugar:  Plain water | Beverages with artificial sweeteners (no caffeine), or low levels of added sugar:  Diet soft drinks  Diet sports drinks  Diet vitamin enhanced-water  Coconut water | | | Beverages with high levels or added sugar; OR with caffeine:  Soft drinks  Sports drinks  Vitamin enhanced water  Energy drinks  Fruit drinks  Slushies  Hot Chocolate  Frappuccino/ Iced Cappuccino  Alcohol | | |

General assumptions made to rank products on FoodMATS and foods and beverages on concession sales

| Dip/dressing is on the side of fruits and vegetables unless otherwise specified.  Dried fruit and fruit sauces are unsweetened unless otherwise specified.  Smoothies are fruit and milk based but contain some added sugar and are always “Less Healthy” unless otherwise specified.  100% juice is always “Less Healthy” due to its high sugar concentration, even though it contains no added sugar  Rice, pasta, bread, pitas, and wraps are non-whole grain and always “Less Healthy” unless otherwise specified.  Oatmeal is without added fat and sugar unless otherwise specified and is always “Most Healthy”.  Granola bars had lower levels of added sugar and is always “Less Healthy” unless otherwise specified.  Cold cereal (no added milk) has high added sugar and is always “Least Healthy” unless otherwise specified. Cold cereal with milk is assessed as a mixed dish.  Pancakes always have high added sugar and fat and are always “Least Healthy” unless otherwise specified.  Cookies and dessert-like baked goods had high fat and sugar and were always “Least Healthy” unless otherwise specified.  Crackers are high in fat and sodium and are always “Least Healthy” unless otherwise specified.  All milk is plain low-fat and always “Most Healthy” unless otherwise specified. Assume all soy and almond milks are sweetened and always “Least Healthy” unless otherwise specified.  Yogurt is flavoured and always “Less Healthy” unless otherwise specified.  Cheese is processed and always “Least Healthy” unless otherwise specified.  Eggs are always “Most Healthy” unless they are in a mayonnaise-based salad.  Nuts and nut butters have low levels of added sugar and salt and are always “Less Healthy” unless otherwise specified.  No condiments are “Most Healthy” since condiments generally are a source of added fat, sugar, or salt.  General “Dip”, “Dipping Sauce” are always “Least Healthy”  “Other foods” are always “Least Healthy” since these are food generally high in 2 added fat, sugar, and salt.  Soft drinks, sports drinks, and vitamin enhanced water, energy drinks are high sugar and are always “Least Healthy” unless otherwise specified. |
| --- |

Assumptions made for ranking "Mixed Dishes with Protein" on concession sales

| - Breakfast items (e.g. “Big Breakfast”, breakfast sandwich, etc.) have eggs. - Breakfast sandwiches/wraps always have cheese. - Soups do not have a protein source unless indicated. - Soup is always “Less Healthy” unless it specifies it is a packaged dry soup mix (which we assume is “Least Healthy”). - Sandwiches do not have vegetables unless it was in the name (e.g. “tomato tuna sandwich”, and that it did have vegetables in the name it satisfied vegetable requirements. - Sandwiches have protein (i.e. analyze as mixed dish with protein), but only have a quality protein if it in the product name (i.e. “wrap” has protein but not a quality protein; “chicken wrap” has protein and we assume it is a quality protein; “grilled chicken wrap” has protein and it is clearly a quality protein; “crispy chicken wrap” has protein but it is a low quality protein. - All pizza has a non-quality protein source (unless it is “cheese pizza”) and it is “Least Healthy” unless the product name or concession audit reveals that it meets the criteria for “Less Healthy” or “Most Healthy”. Cheese pizza is always “Least Healthy”. |
| --- |

1. Protein source is any meat or alternative product. It does not include milk and alternative products. [↑](#footnote-ref-1)
2. Assume all breads, buns, wraps, etc. are not whole grain unless specified in the sales data or the concession audit. “Brown bread” is counted as whole-grain. [↑](#footnote-ref-2)
3. Quality proteins include baked, broiled, boiled, grilled, or roasted fresh or frozen meats; or legumes, eggs, nut butters. It does not include milk and alternatives (milk, cheese, yogurt, etc.). Quality proteins cannot be deep-fried or breaded at any point and cannot be high fat/salt meats (ham, pastrami, salami, pepperoni, corned beef). [↑](#footnote-ref-3)
4. Vegetable serving equals ½ cup (125ml) of fresh, frozen, canned non-leafy vegetables or 1 cup (250ml) of leafy vegetables. [↑](#footnote-ref-4)
5. Added fat for entrees include any presence of high fat sauce (cheese sauce), ingredients (cheese, bacon, avocado, mayonnaise based salad), or sides (French fries, onion rings); it does not include butter, margarine, or mayonnaise spread on sandwiches. Added fat for vegetable-based entrée sized salads represent having more than 2 of the following: full fat dressing not on side, avocado, bacon, cheese, croutons, egg, fried noodles, crushed tortilla chips, nuts, olives, pesto, sausage, pepperoni, salami, bologna, pastrami, high fat lunch meat, or sour cream (meats are counted if they are in addition to the main protein). [↑](#footnote-ref-5)
6. Healthy side includes vegetables, fruit, or other “Most Healthy” foods. [↑](#footnote-ref-6)
7. Always includes: pizza, hamburgers, hot dogs, beef tabos, pasta and cheese, grilled cheese [↑](#footnote-ref-7)
